# Genomic basis of broad host range and environmental adaptability of *Rhizobium tropici* CIAT 899 and *Rhizobium* sp. PRF 81 which are used in inoculants for common bean (*Phaseolus vulgaris* L.)

**DOI:** 10.1186/1471-2164-13-735

**Published:** 2012-12-27

**Authors:** Ernesto Ormeño-Orrillo, Pâmela Menna, Luiz Gonzaga P Almeida, Francisco Javier Ollero, Marisa Fabiana Nicolás, Elisete Pains Rodrigues, Andre Shigueyoshi Nakatani, Jesiane Stefânia Silva Batista, Ligia Maria Oliveira Chueire, Rangel Celso Souza, Ana Tereza Ribeiro Vasconcelos, Manuel Megías, Mariangela Hungria, Esperanza Martínez-Romero

**Affiliations:** 1 Centro de Ciencias Genómicas, Universidad Nacional Autónoma de México, Cuernavaca, Morelos, Mexico; 2 Embrapa Soja, C. P. 231, Londrina, Paraná, 86001-970, Brazil; 3 Laboratório Nacional de Computação Científica (LNCC), Avenida Getúlio Vargas 333, Petrópolis, Rio de Janeiro, Brazil; 4 Universidad de Sevilla, Apdo Postal 874, Sevilla, 41080, Spain; 5 Universidade Estadual de Londrina, Cx. Postal 60001, Londrina, Paraná, 86051-990, Brazil

**Keywords:** Nodulation, Nitrogen fixation, Plant-microbe interactions, Antimicrobial resistance

## Abstract

**Background:**

*Rhizobium tropici* CIAT 899 and *Rhizobium* sp. PRF 81 are α-Proteobacteria that establish nitrogen-fixing symbioses with a range of legume hosts. These strains are broadly used in commercial inoculants for application to common bean (*Phaseolus vulgaris*) in South America and Africa. Both strains display intrinsic resistance to several abiotic stressful conditions such as low soil pH and high temperatures, which are common in tropical environments, and to several antimicrobials, including pesticides. The genetic determinants of these interesting characteristics remain largely unknown.

**Results:**

Genome sequencing revealed that CIAT 899 and PRF 81 share a highly-conserved symbiotic plasmid (pSym) that is present also in *Rhizobium leucaenae* CFN 299, a rhizobium displaying a similar host range. This pSym seems to have arisen by a co-integration event between two replicons. Remarkably, three distinct *nodA* genes were found in the pSym, a characteristic that may contribute to the broad host range of these rhizobia. Genes for biosynthesis and modulation of plant-hormone levels were also identified in the pSym. Analysis of genes involved in stress response showed that CIAT 899 and PRF 81 are well equipped to cope with low pH, high temperatures and also with oxidative and osmotic stresses. Interestingly, the genomes of CIAT 899 and PRF 81 had large numbers of genes encoding drug-efflux systems, which may explain their high resistance to antimicrobials. Genome analysis also revealed a wide array of traits that may allow these strains to be successful rhizosphere colonizers, including surface polysaccharides, uptake transporters and catabolic enzymes for nutrients, diverse iron-acquisition systems, cell wall-degrading enzymes, type I and IV pili, and novel T1SS and T5SS secreted adhesins.

**Conclusions:**

Availability of the complete genome sequences of CIAT 899 and PRF 81 may be exploited in further efforts to understand the interaction of tropical rhizobia with common bean and other legume hosts.

## Background

The symbiotic relationship between legumes and nitrogen fixing-bacteria, commonly known as rhizobia, has been the subject of practical and basic studies for over 120 years. Lately, a renewed interest in the topic has been observed due to its key role in agriculture sustainability, in lowering costs for the farmers, in improving soil fertility, and in the mitigation of greenhouse-gas emissions.

The rhizobia-legume symbiosis starts with a finely tuned molecular dialogue between the partners. Specific signals released by the legume, mainly flavonoids [1], are perceived by the rhizobium that, in response, produces lipochitin oligosaccharides, the Nod factors, that in turn elicit the formation of specialized organs, the root nodules [2]. Rhizobia enter the legume roots and colonize the developing nodules where they differentiate into bacteroids that fix atmospheric nitrogen [3]. In addition, a variety of other bacterial systems are required for root colonization, effective nodulation and nitrogen fixation, including surface polysaccharides and secretion systems [3,4].

Nitrogen fixation is an ancient prokaryotic trait that predates plant evolution [5], as evidenced by the distribution of diazotrophs in a broad range of non-phylogenetically related bacteria [6]. On the other hand, rhizobia-induced nodulation and the corresponding nodulation (*nod*) genes had a more recent origin, arising with the evolution of the host legumes [7]. *nod* and *nif* genes are contained in symbiotic plasmids or symbiotic islands. Rhizobial symbiotic compartments are enriched in mobile genetic elements, and show evidence of gene acquisition by lateral transfer [8,9]. This dynamic evolution is probably driven by selective pressures to nodulate a range of legume hosts.

The best studied rhizobium-legume symbiotic models are those involving *Sinorhizobium* (=*Ensifer*) *meliloti*-alfalfa (*Medicago sativa* L.) and *Bradyrhizobium japonicum-*soybean *Glycine max* (L.) Merr.], and significant progress has been achieved by sequencing the genomes of the bacterial partners [10,11]. The genome sequence of *Rhizobium etli* CFN 42 shed light on its symbiotic relationship with common bean (*Phaseolus vulgaris* L.), the most important legume for direct human consumption in undeveloped and developing countries. However, poor understanding prevails with respect to other important common bean-nodulating symbionts of tropical acid soils, such as *Rhizobium* strains CIAT 899 and PRF 81 [12].

CIAT 899, isolated from a common-bean nodule in Colombia, is the type strain of *Rhizobium tropici*[13]. Originally, *R. tropici* comprised two phenotypically distinct groups, named type A and type B, but recently a new species, *Rhizobium leucaenae*, has been proposed to accommodate the type A strains, with strain CFN 299 selected as the representative of the species [14]. Strain PRF 81 was isolated from a common-bean nodule collected in the State of Parana, Brazil; it is phylogenetically related to *R. tropici* and *R. leucaenae*[15]. PRF 81 has been described as intermediate in phenotypic characteristics between *R. tropici* and *R. leucaenae*, although it has been suggested to be more closely related—phenotypically and phylogenetically—to the former species [15].

CIAT 899 and PRF 81 are promiscuous rhizobia with host ranges that include several species of the three legume subfamilies [15]. Several wild tropical legumes are the natural hosts of *R. tropici* and *R. leucaenae* strains [16], and these rhizobia gained access to common bean nodules when the legume was introduced into tropical regions [12]. The genetic determinants of the broad host range of *R. tropici* are presently unknown, although the capacity of certain strains, like CIAT 899, to produce a wide range of Nod factor structures [17] surely contributes to this phenotype. In addition to its association with legumes, CIAT 899 is also a proficient maize rhizospheric and endophytic colonizer, with the capacity to promote plant growth [18].

CIAT 899 and PRF 81 have been identified as highly effective in fixing N_2_ with Andean and Mesoamerican common-bean genotypes, competitive against indigenous rhizobia, genetically stable, and adapted to stressful tropical conditions such as acidic soils and high temperatures [13,15,19,20]. These properties resulted in the inclusion of both strains in commercial inoculants in Brazil [21,22], and in some countries in Africa. For CIAT 899, the genetic determinants of growth at high temperatures and low pH that have been reported [23-27] do not fully explain the ability of this strain to withstand such stressful conditions. In addition, PRF 81 outcompetes CIAT 899 in co-inoculation experiments for bean nodulation under greenhouse conditions [15] and in field trials in acid soils [21]; however, the basis of this differential competitiveness remains unexplored, as well as the possible repertoire of genes related to the competitive ability of both strains when challenged with indigenous rhizobia.

CIAT 899 and other *R. tropici* strains are more resistant to several antimicrobial compounds and heavy metals in comparison to other common bean-nodulating rhizobia, such as *R. etli* and *R. leucaenae*[14,28]. *R. tropici* strains also show strong resistance to pesticides used in agriculture, such as the fungicides Thiram and Captan, that can diminish the survival of rhizobial inoculants when applied to seeds [29,30].

The aim of this study was to compare the genome of *R. tropici* CIAT 899 with that of *Rhizobium* sp. PRF 81, with special reference to their symbiotic plasmids and all possible determinants of resistance to stressful conditions and antimicrobial compounds. The sequence of the symbiotic plasmid of *R. leucaenae* CFN 299 was also obtained and analyzed. Possible mechanisms of broad host range and symbiotic plasmid evolution were found, as well as features that may explain the basis of the environmental stress tolerance and antimicrobial resistance of these strains.

## Results and discussion

### General characteristics of CIAT 899 and PRF 81 genomes

*R. tropici* CIAT 899 had a 6,686,337-bp genome composed of a chromosome and three plasmids with *rep*-type replication systems. General features and statistics of the genome are presented in Table 1. The 3,837,060-bp chromosome contained all three ribosomal operons. The plasmid sizes were in general agreement with previously reported estimates based on Eckhardt gels [31], except for the 2,083,197-bp megaplasmid (pRtrCIAT899c) which was somewhat larger than expected. The second largest replicon (549,467 bp) was the symbiotic plasmid (pSym). The 216,610-bp, smallest plasmid (pRtrCIAT899a) and the pSym had lower G + C-content values than the genome average (Table 1).

**Table 1:** Genome size and number of predicted genes for CIAT 899 and PRF 81

	**CIAT 899**					**PRF 81**^**†**^					
	**Chr**	**pC**	pB^*^	**pA**	**Genome**	**Chr**	**pD**	pC^*^	**pB**	**pA**	**Genome**
Size (Mb)	3.8	2.08	0.55	0.22	6.69	3.75	2.46	0.52	0.18	0.14	7.08
G + C (%)	59.9	59.4	57.6	58.6	59.5	60.4	59.8	57.5	59.1	59.1	59.9
Number of predicted genes	3734	1905	500	212	6351	3479	2093	478	149	132	6331
CDS	3672	1905	500	212	6289	3419	2093	478	149	132	6271
tRNA	53	-	-	-	53	51	-	-	-	-	51
rRNA	9	-	-	-	9	9	-	-	-	-	9

^*^ Symbiotic plasmid.

^†^ For PRF 81, the size and G + C content of the chromosome, pD and pC are estimates.

Sequencing of *Rhizobium* strain PRF 81 resulted in a high-quality draft genome with an average 17.4-fold coverage, and distributed in 103 contigs with an N50 size of 246 kb. The estimated genome size was 7.08 Mb. It was previously shown that PRF 81 has four plasmids [32]. Two contigs were found to represent the complete sequences of the two smallest plasmids pPRF81a and pPRF81b (Table 1). Given that chromosomes in the Rhizobiaceae are conserved [33], an alignment of the PRF 81 contigs to the genomes of CIAT 899 and other *Rhizobium* strains allowed the identification of 23 putative chromosomal contigs of 3.76 Mb in total size. Interestingly, seven contigs of PRF 81 mapped with a very high level of identity to the CIAT 899 pSym and were 520 kb in total size, a value close to the estimated size of the PRF 81 pSym (pPRF81c) [15]. The remaining contigs (2.49 Mb in total size) must represent sequences from the PRF 81 megaplasmid (pPRF81d) that has an estimated size of 2.4 Mb, or repeated sequences in the genome.

Putative functions could be assigned to around 67% of the coding DNA sequences (CDS) predicted for each strain. The chromosomes coded for most genes assigned to functionally important classes such as central intermediary metabolism, cellular processes and DNA metabolism. No essential genes were found in plasmids. However, it is noteworthy that megaplasmids from both strains encoded about 45% of all the transport capacity of the genome, 37% of the regulatory functions and 34% of energy-related metabolism.

### Phylogeny and whole genome relationships between CIAT 899 and PRF 81

**Figure 1:**
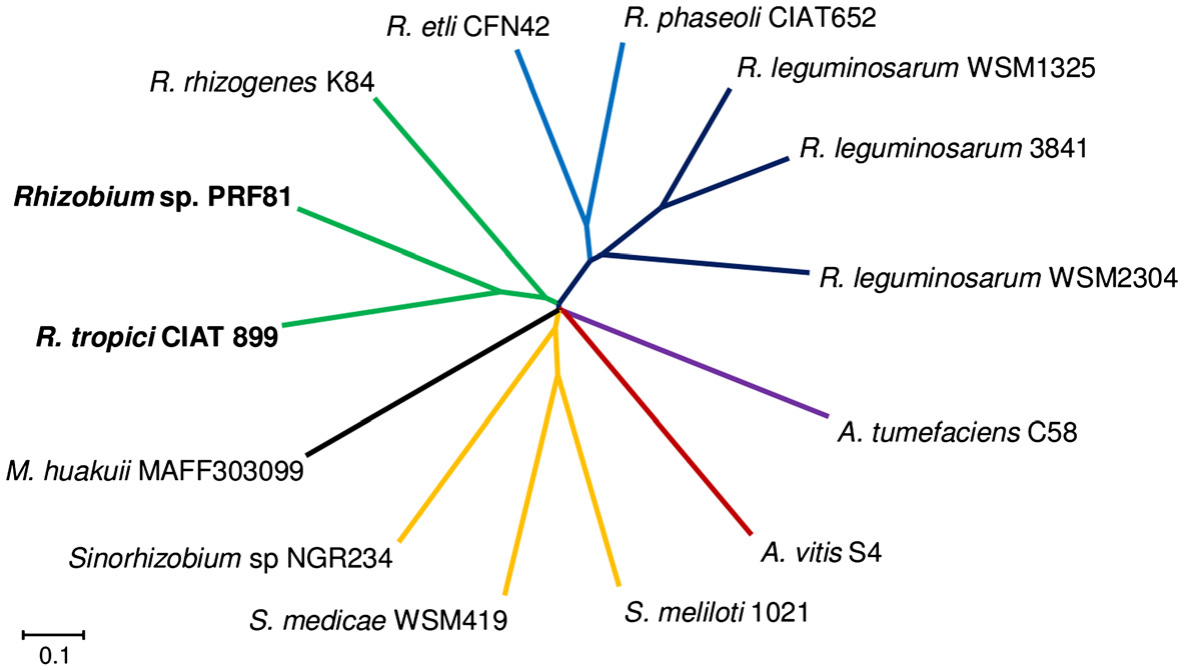
**Whole genome relationships between *Rhizobium tropici *CIAT 899, *Rhizobium *sp. PRF 81 and other Rhizobiaceae strains.** Based on MUMi genome distances.

*R. tropici* forms a clade with *R. leucaenae* (formerly *R. tropici* type A), *Rhizobium multihospitium, Rhizobium lusitanum, Rhizobium miluonense* and *Rhizobium rhizogenes*, in phylogenies constructed with the 16S rRNA and several housekeeping genes [14,34]. The only other representative of this clade with a sequenced genome is *R. rhizogenes* K84 [35-37]. We determined the relationships between the genomes of CIAT 899, PRF 81 and other Rhizobiaceae genomes using a dendrogram constructed with the genomic-distance index MUMi [38]. As shown in Figure 1, CIAT 899 and PRF 81 were the most closely related strains, and they had K84 as their sister taxon, which is consistent with the close relationship between the former strains and *R. rhizogenes*. The dendrogram also correctly depicts accepted phylogenetic relationships clustering strains from the same species (*Rhizobium leguminosarum*), as well as strains from closely related species like *R. etli* and *Rhizobium phaseoli*, or *S. meliloti* and *Sinorhizobium medicae*. As expected, due to their close relationship, CIAT 899 and PRF 81 shared a higher number of orthologous genes with each other than with any other sequenced rhizobia or agrobacteria (Additional file 1). Phenotypic and phylogenetic analyses previously reported by us suggest that, although close to CIAT 899, PRF 81 may be divergent enough to be considered as a different species [34]. When a digital DNA-DNA hybridization methodology [39] was performed, PRF 81 shared only 52% of its genome sequence with CIAT 899, suggesting that wet-lab hybridization will be lower than the 70% threshold required for inclusion in the species *R. tropici*[40].

**Figure 2:**
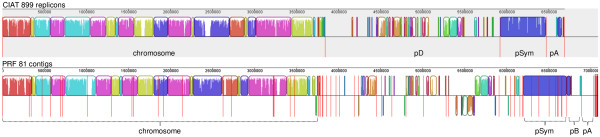
**Conservation of synteny between the genomes of *Rhizobium tropici *CIAT 899 and *Rhizobium *sp. PRF 81.** Replicons/contigs are separated by vertical red lines. Syntenic regions are outlined and shown with the same color. PRF 81 contigs were ordered using MAUVE.

Synteny, evaluated at the contig level, was found between CIAT 899 and PRF 81 chromosomes and pSyms (Figure 2). Scattered syntenic gene clusters were found between their megaplasmids. These clusters included the septum formation *minCDE*, histidine degradation *hutIHU,G*, and protocatechuic acid degradation *pcaGHCDQRIJF* genes, all of them previously found to be conserved in secondary replicons of bacteria [35]. Recently, the term chromid was proposed for secondary replicons showing chromosome-like features but plasmid-type replication systems [41]. The megaplasmids from both strains conformed to the characteristics defined for chromids, such as having similar G + C contents as the chromosome (Table 1) and harboring core genes usually found in chromosomes. For example, both megaplasmids encoded for thiamin and cobalamin (vitamin B12) biosynthesis.

It has been proposed that a “chromid often arises at the origin of a new genus” by the migration of essential or important genes to a plasmid [41]. This may explain why a large proportion of genes contained in chromids are conserved within a genus [41]. In the case of CIAT 899 and PRF 81, 47% of the genes contained in the CIAT 899 chromid had orthologues located in putative chromid contigs of PRF 81. After genus emergence and species divergence, chromids may evolve differently. For example, in the *R. etli*/*R. leguminosarum* lineage, it seems that the original chromid suffered events of co-integration and resolution leading to its partition into three different replicons, while within the *R. tropici* lineage it was maintained as a single replicon. In contrast to CIAT 899 and PRF 81 chromids, those of *R. etli* and *R. leguminosarum* are highly syntenic [42]. The lack of chromid gene order conservation between CIAT 899 and PRF 81 may indicate that these strains have a longer history of divergence in comparison to *R. etli* and *R. leguminosarum*, again indicating that they belong to different species.

### A conserved symbiotic plasmid defining a novel symbiovar: tropici

**Figure 3:**
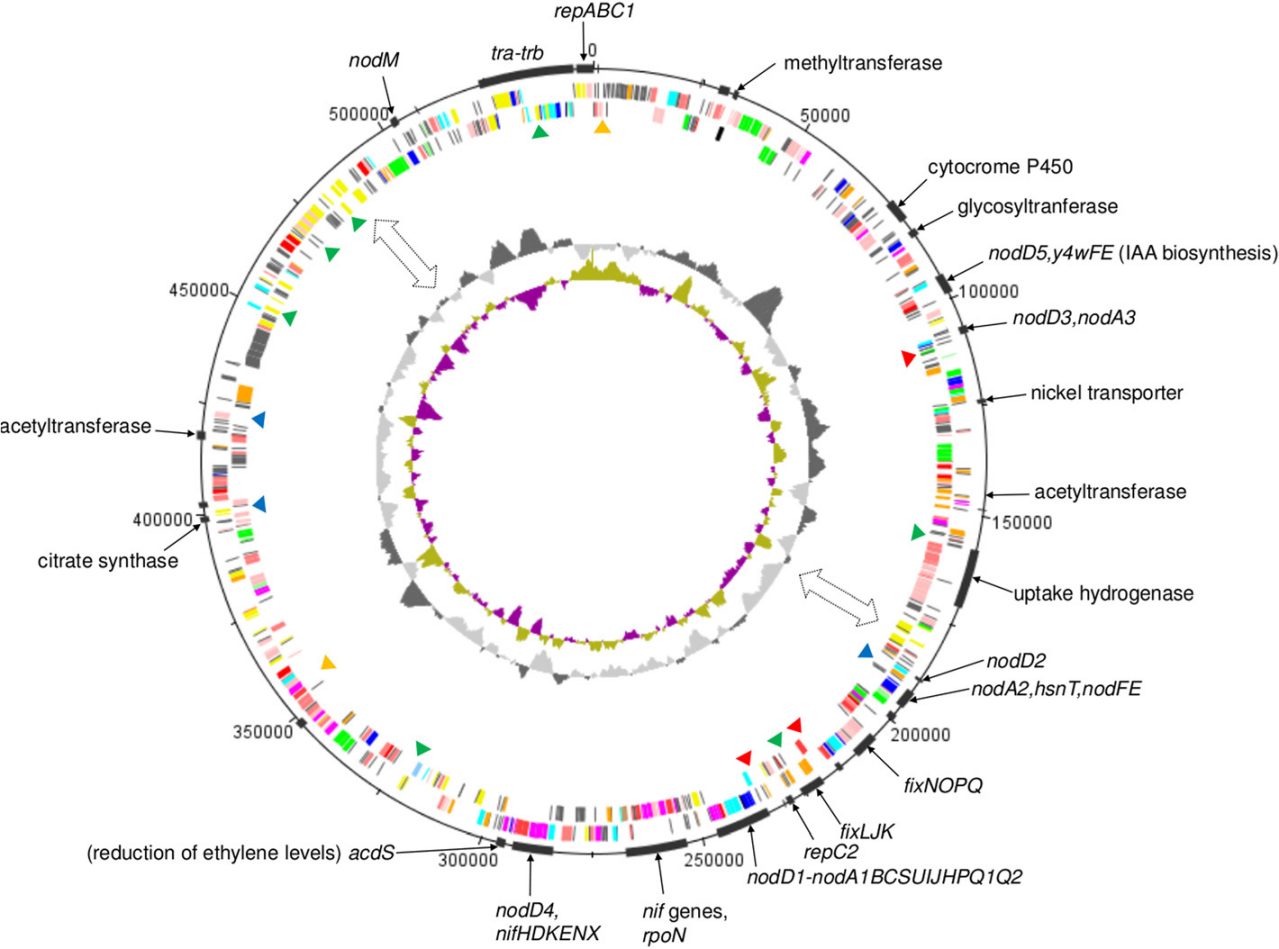
**Representation of the tropici symbiotic plasmid.** Circles from innermost to outermost indicate: GC skew, GC content, location of ISs differentially present in the three strains analyzed (green, only CIAT 899; blue, only in PRF 81; orange, only in CFN 299; red, CIAT899 and PRF 81), genes in reverse orientation, genes in forward orientation, and locations of some of the genes or functions mentioned in the text. Double-headed arrows indicate the position of IS-rich regions and junctions of two regions with different GC content representing two putative ancestral plasmids that gave rise to this pSym.

CIAT 899 and PRF 81 are promiscuous rhizobia sharing a broad host range with *R. leucaenae* CFN 299 (Additional file 2). Previous studies reported that these three strains produce similar Nod factors [43] and have almost identical sequences in analyzed symbiotic genes. To compare the symbiotic gene repertoire of these strains, we sequenced the CFN 299 pSym. This plasmid was transferred to a plasmid-less *Agrobacterium* strain and purified. A combination of Sanger and Solid sequencing was used, resulting in 27 contigs with 20-fold coverage, representing the almost complete CFN 299 pSym. We found that the three strains have a conserved and syntenic pSym, hereafter designated as the “tropici symbiotic plasmid” (Figure 3). The level of sequence identity between the three plasmids was very high (≥99.9% at the contig level), therefore all gene products were 100% identical at the amino acid level. This high sequence conservation likely indicates a recent dissemination of the tropici pSym. The only major differences between the three pSyms were the presence or absence of certain insertion sequences (ISs) (Figure 3). We have previously demonstrated that several ISs located in the pSym of CFN 299 are active at transposition [44].

Two replication systems were found in the tropici pSym, one having a set of *repABC1* genes and the other only *repC2*. The highest similarities of RepC2 (74–77%) were with homologues located in *S. meliloti* and *Sinorhizobium fredii* plasmids. RepC1 also showed high similarities (82–87%) with sinorhizobial genes and also with RepCs from *Rhizobium* and *Agrobacterium*. Interestingly, two regions showing different G + C contents could be recognized in this plasmid, a 236-kb segment harboring the *repABC1* genes showed 59%, whereas the other 291-kb segment harboring the *repC2* gene showed 57%. The two regions are connected by 19.7-kb and 7.2-kb regions constituted by ISs and integrase genes. Thus, the pSym may have arisen by the co-integration of two plasmids. The largest region contained the core genes required for nodulation and nitrogen fixation, and probably represent the original pSym. The other region contained genes that could have improved the symbiotic or associative abilities, such as *nodM*, an uptake hydrogenase, and genes for the biosynthesis of plant hormones. The tropici pSym possessed the majority of all IS transposases present in the genomes of CIAT 899 and PRF 81. Members of the IS*66* family were the most abundant ISs as also have been observed in symbiovar phaseoli plasmids [45]. Shared ISs between plasmids may promote co-integration events through homologous recombination.

The term symbiovar, recently proposed to replace the term biovar, is useful to designate symbiotic variants that exist in different rhizobial species [46]. Symbiovars are defined based on legume-host range and on symbiotic gene sequences. Therefore, it seems reasonable to propose that CIAT 899, CFN 299 and PRF 81 belong to the same symbiovar that here we designate as tropici. *R. lusitanum* P1-7, also ascribed to the *R. tropici* clade, has *nodD* and *nifH* genes resembling those in CIAT 899 [47] and induces effective nodulation in *Leucaena* sp.; therefore, it should also be ascribed to symbiovar tropici. The symbiovar tropici pSym shares an analogous history with symbiovar phaseoli pSym in the sense that a highly conserved plasmid has spread and is maintained in several different *Rhizobium* species [46].

### Nodulation genes

Using different techniques, similar Nod factor structures have been attributed to CFN 299 [48], CIAT 899 [49] and PRF 81 [15]. The tropici pSym gene cluster *nodD-ABCSUIJ-HPQ*, described in previous studies [49,50], is able to direct the synthesis of Nod factors and to confer nodulation ability when transferred to a non-nodulating strain [51]. *nodC* directs the synthesis of the Nod factor-backbone chitin oligosaccharide structure that is deacetylated, acylated, methylated, carbamoylated and sulfated by the products of *nodB*, *nodA*, *nodS*, *nodU* and *nodH*, respectively [49,50], and exported by an ABC-type transporter encoded by *nodIJ*[52]. The activated sulfate donor used by NodH is produced by the *nodPQ* products. Unexpectedly, the two functional domains of NodQ, sulfate adenylyltransferase and adenylylsulfate kinase, previously reported to be carried on a single polypeptide [49], were found in this study to be encoded in distinct but overlapping genes, designated as *nodQ1* and *nodQ2*, respectively.

**Figure 4:**
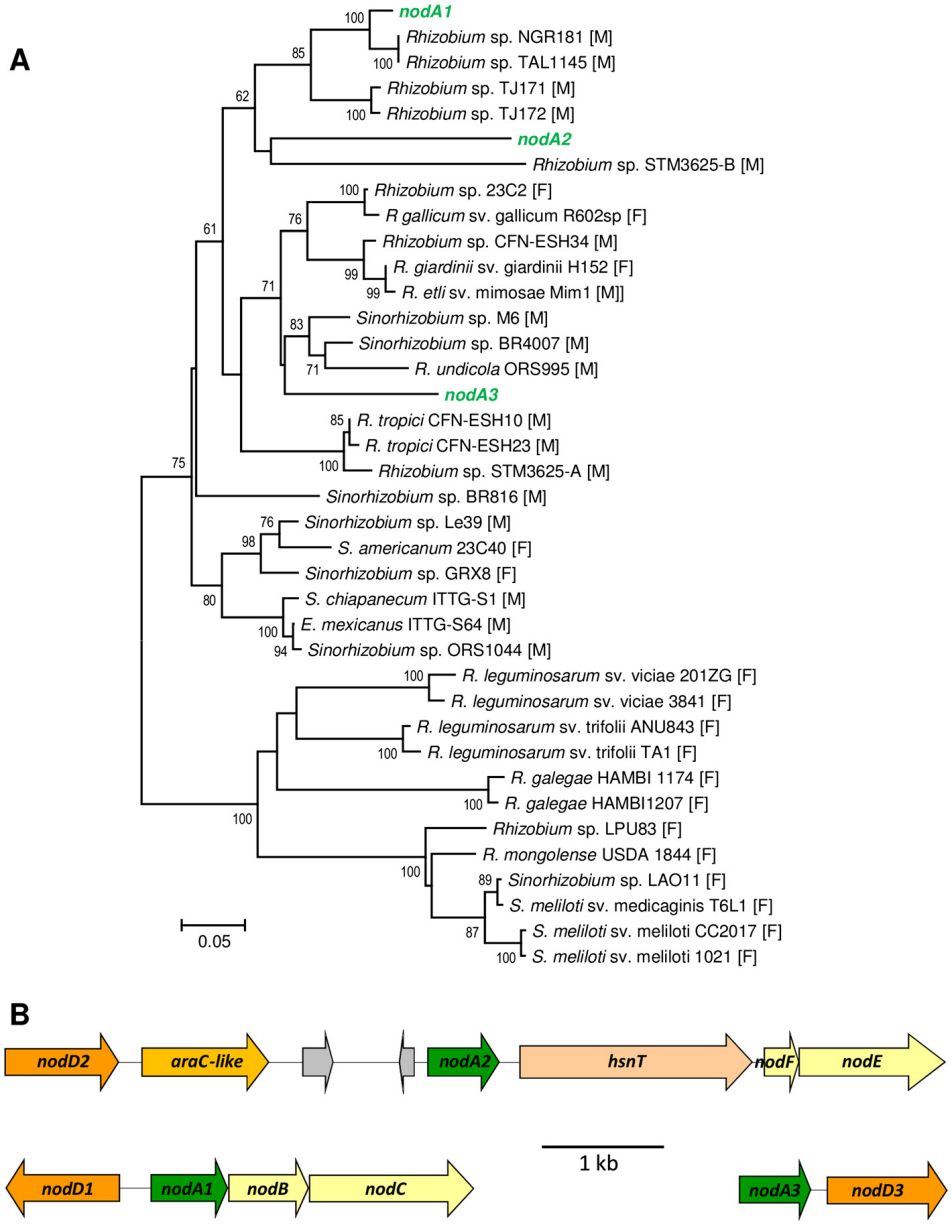
**Three divergent *nodA *genes in the tropici symbiotic plasmid. A**, Maximum likelihood phylogeny of *nodA* genes. Genes from the tropici pSym are shown in green. Host-legume subfamily affiliation is indicated within brackets (M, Mimosoideae; F, Faboideae). Strain STM3625 possesses two *nodA* genes. **B**, Gene neighborhood of the three *nodA* genes. Genes represented with gray color are hypothetical.

**Figure 5:**
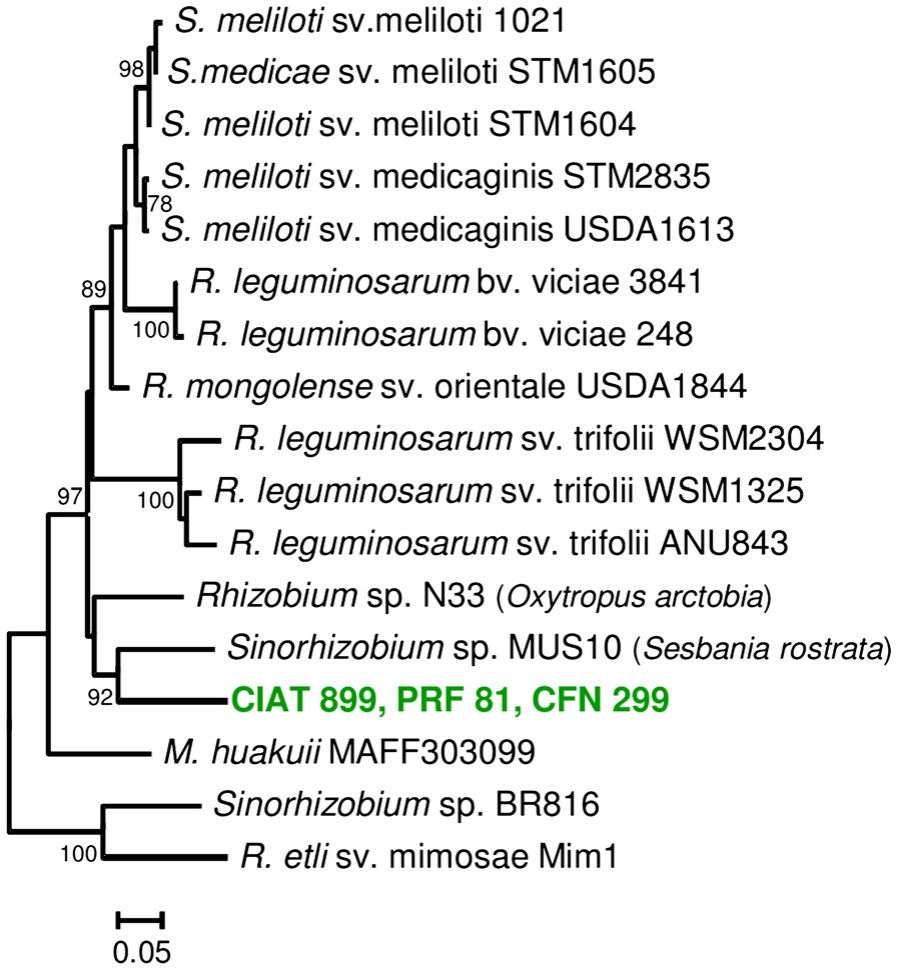
**Maximum likelihood phylogeny of NodE proteins.** Host legume of some strains is indicated within parenthesis.

It has been proposed that the nature of the Nod factor acyl group attached by NodA can contribute to the determination of host range [53]. Interestingly, we found that, in addition to the *nodA* gene located adjacent to *nodBC*, two additional *nodA* genes are present in the tropici symbiotic plasmid. The amino acid identity between the three *nodA* products ranged from 72 to 78%, suggesting that they are not functional redundant copies. Phylogeny of the three NodA proteins indicated that they cluster within a large group of sequences from rhizobia isolated mostly from tropical Mimosoideae legumes (Figure 4A), as previously noted for the *R. leucaenae* CFN 299 *nodA* gene [54]. Mimosoideae rhizobia may have broader host ranges including Papilionoideae and Faboideae legumes as is known for CIAT 899, PRF 81 and CFN 299. The two additional *nodA* genes from the tropici symbiotic plasmid, named *nodA2* and *nodA3*, did not have close homologues in the databases (Figure 4A). The copy named *nodA2* was part of a gene cluster including *nodFE* and *hsnT* (Figure 4B). The acyl carrier protein NodF and the β-acetoacetylsynthase NodE are required for the biosynthesis of α,β-unsaturated fatty acids present in Nod factors produced by some rhizobia and are determinants of host specificity [55]. *hsnT* encodes an uncharacterized acyltransferase that is unrelated to NodA proteins. It seems likely that the tropici pSym *nodA2-hsnT-nodEF* gene cluster directs the biosynthesis and incorporation of α,β-unsaturated acyl chains that have not been observed to date in Nod factors produced by CIAT 899 or CFN 299. The NodE protein encoded in the tropici pSym is most similar (Figure 5) to the corresponding protein of *Sinorhizobium* sp. strain MUS10, a symbiont of the tropical legume *Sesbania rostrata*[56]. Neighbor genes of the third copy, *nodA3*, did not provide any indication of its acyl chain specificity. Additional and divergent *nodA* genes likely expand the diversity of Nod factor acyl chains and may contribute to widening the host range in these strains.

LysR-family transcriptional regulators encoded by *nodD* genes mediate activation of *nod*-gene expression in response to plant-produced signal molecules (mainly flavonoids) [57,58]. Different NodD proteins vary in their responses to different sets of flavonoids and some strains have several *nodD* genes [3]. In CIAT 899, five *nodD* genes have been described [59] and here we confirmed their presence in the sequenced tropici pSyms. When *nodD1*, the copy adjacent to *nodABC*, is transferred to *R. etli* and *R. leguminosarum* sv. trifolii, it confers the ability to nodulate *L. leucocephala* and *P. vulgaris*, respectively [60]. Of the five *nodD* genes of CIAT 899, *nodD1* is mainly responsible for *nod*-gene activation with various purified flavonoids, and root or seed exudates from common bean, *L. leucocephala*, and *Macroptilium atropurpureum*[59,61]. In agreement, a *nodD1* mutant is unable to nodulate *L. leucocephala* and *M. atropurpureum*, and shows severely diminished nodulation of common bean [61]. This evidence suggests that the remaining *nodD* copies fulfill accessory roles in nodulation, at least with the three hosts tested to date. *nodD2* and *nodD3* were in close vicinity to *nodA2-hsnT-nodEF* and *nodA3*, respectively (Figure 4B), and may be responsible for their activation in response to signals from plants that require Nod factors with specialized acyl chains. An AraC-family transcriptional regulator gene was found located adjacent to *nodD2* and may also participate in *nodA2* regulation. Interestingly, *nodD4* and *nodD5* were in close proximity to gene clusters required for plant-hormone biosynthesis and nitrogen fixation, respectively, as discussed below.

The tropici pSym did not harbor *nolO* homologues that in other rhizobia are responsible for carbamoylation at the 3’ (or 4’) position of the non-reducing terminal glucosamine, indicating that Nod factors produced by our strains are carbamoylated only at the 6’ position by NodU. Glycosyl residues attached at the reducing end of the CIAT 899 Nod factors include mannose [49] and fucose or methyl fucose, the latter two present only after growth under saline stress [62]. The mannosyl substitution has been described only in CIAT 899 and the genetic determinants are unknown. Fucosyl residues are present in a range of rhizobial strains harboring *nodZ genes*, and they can be methylated by the *noeI* gene product. We did not find homologues to *nodZ* or *noeI* in the tropici pSym, nor elsewhere in the CIAT 899 and PRF 81 genomes. A putative glycosyltransferase and methyltransferase were encoded in the tropici pSym though they are not related to any described *nod* gene.

A *nodM* homologue was identified in the tropici pSym. NodM is a paralogue of the housekeeping enzyme glucosamine synthase, GlmS, and seemingly provides adequate amounts of the unit blocks used by NodC for Nod factor biosynthesis. Symbiotic plasmids or islands in *R. leguminosarum*, *S. meliloti*, *S. medicae* and *Mesorhizobium* spp. also harbor paralogous glucosamine synthases. NodM is required for efficient nodulation of some hosts [63].

Nodulation factors produced by CIAT 899 are acetylated at the non-reducing end, which, in *R. leguminosarum* and *S. meliloti*, is carried out by the pSym *nodL* gene product [3]. Although two acetyltransferase genes were located in the tropici pSym, they did not show significant similarities to *nodL*; besides, one of those genes was disrupted by an IS in CIAT 899, but not in PRF 81 or CFN 299. On the other hand, a *nodL*-like gene located in the chromosomes of CIAT 899 and PRF 81 encodes a putative acetyltransferase 63% similar to *R. leguminosarum* 248 NodL, encoded in its pSym [64]. Genes *noeJ* and *noeK*, *noeL* (=*gmd*) and *nolK* (=*fcl*), required for biosynthesis of the activated sugar donors GDP-D-mannose and GDP-L-fucose, respectively—usually necessary for the biosynthesis of common surface polysaccharides [30]—were encoded in the chromosomes of CIAT 899 and PRF 81.

CIAT 899 and PRF 81 chromosomes also encoded proteins that are highly similar (91%) to the transcriptional repressor NolR of *R. leguminosarum*. NolR-mediated repression of *nod*-gene expression after induction by NodD is required for optimal nodulation of some hosts [65]. Inspection of rhizobial genome sequences revealed that *nolR* homologues are located in chromosomes or chromids.

### Nitrogen-fixation genes

The tropici pSym harbored a set of nitrogen-fixation (*nif*/*fix*) genes similar to those in other tropical microsymbionts, comprising a higher number of genes in comparison to those present in temperate rhizobial species [66]. *nif*/*fix* genes were organized in four clusters in the tropici pSym. The first cluster was preceded by *nodD4* and contained *nifHDK* coding for the nitrogenase structural components, *nifEN* whose products function as scaffolds for FeMo-cofactor assembly, and *nifX* coding for a FeMo-cofactor binding protein [6]. These *nif* genes showed the highest similarity to the corresponding genes of *R. etli* and *M. loti*. It is noteworthy that, in no other rhizobial species, a *nifHDKEN* cluster is preceded by a regulatory *nodD* gene, and it remains to be established if the expression of these *nif* genes is dependent on this transcriptional regulator and is increased by specific flavonoids. The second gene group, located after the main *nod*-gene cluster, included *nifTZ, fdxN, fixN, nifBA, fixXCBA, nifWSU* and *nifQ*, which also showed the highest similarity to homologues in *M. loti* and *R. etli*. Although the function of some of these genes is not known (*nifT*, *nifW*), the remaining *nif* genes are involved in nitrogenase maturation (*nifZ*), biosynthesis of the FeMo-cofactor (*nifB*, *S*, *U* and *Q*), and transcriptional activation of *nif* genes (*nifA*) [67]. The *fdxN* and *fix* genes present in this cluster are involved in transfer of electrons to nitrogenase [68]. A distinct gene cluster included the *fixNOQP* operon encoding the cbb3-type terminal symbiotic cytochrome oxidase components, and *fixGHIS* whose products are thought to be involved in copper uptake and metabolism required for the terminal oxidase [69]. These *fix* genes showed strong similarities to the corresponding gene clusters in *R. leguminosarum* and *R. etli* genomes. The *fixLJ* and *fixK* genes, encoding regulatory elements that control the expression of the terminal oxidase under microaerobic conditions, were adjacent to each other in the pSym and showed higher identities with *S. meliloti* genes than with homologues from other *Rhizobium* strains. In CIAT 899, an IS*21* was found inserted between *fixLJ* and *fixK*, although it did not alter the coding sequences. Affiliation of the *nif*/*fix* genes to different microorganisms indicates a high contribution of gene recruitment by horizontal transfer to the genesis of the tropici pSym. This observation is supported by the proximity of mobile genetic elements to several of the described gene clusters.

In contrast to symbiovar phaseoli pSym that has three *nifH* copies, a unique *nifH* gene is present in the tropici symbiotic plasmid as previously reported [13]. More than one *nifH* gene is present in *Sinorhizobium* sp. NGR 234, *Azorhizobium caulinodans* ORS 571, and *Bradyrhizobium* sp. BTAi1 and ORS278. Multiple gene copies can represent hot spots for recombination and genomic rearrangements [70], in some cases leading to loss of symbiotic properties [71]. On the other hand, repeated sequences can promote the generation of symbiotic amplicons where the number of *nod* or *nif* genes can be increased [72].

### An inactive uptake-hydrogenase gene cluster in the tropici pSym

Uptake hydrogenases recycle part of the energy spent in the N_2_-fixation process through the oxidation of hydrogen (H_2_) that is obligatorily produced by the nitrogenase and consumes at least 25% of the reducing power invested in N_2_ fixation. Particularly in the 1970s and 1980s, searches for symbiotic partners expressing uptake hydrogenase were aimed at increasing plant yields by improving the energetic efficiency of the nitrogen-fixation process [73,74]. Among fast-growing rhizobia, only some strains of *R. leguminosarum*, *R. tropici* and *Astragalus* symbionts express hydrogenase activity [75]. In CIAT 899 and CFN 299, positive hybridization signals are observed with a probe containing hydrogenase genes; however, both strains have Hup^–^ phenotypes [76]. Here we confirmed that the tropici pSym carries genes encoding an uptake hydrogenase (Figure 3) showing similarity (~70%) to *R. leguminosarum* homologues. Nevertheless, *hupH* and *hupJ* are truncated, and *hupE*, *hupG* and *hupI* are absent. The precise functions of *hupGHIJ* are not known, but they are required for maturation of the HupS hydrogenase subunit, and their combined absence or alteration in the tropici pSym likely explain the Hup^–^ phenotype reported for CIAT 899 and CFN 299 [77]. The other missing gene, *hupE*, encodes a Ni transporter [78]. A *hupE*-like gene was located 31 kb away from the hydrogenase cluster in the tropici pSym, and CIAT 899 and PRF 81 chromosomes carried an additional *hupE*-like transporter. Introduction of a *R. leguminosarum hupE*-defective hydrogenase gene cluster under the control of a *fixJ* promoter induces only a weak Hup^+^ phenotype in CIAT 899 bacteroids [78], suggesting that the alternative Ni transporters could not fully replace the function of *hupE*.

### Cell-surface polysaccharides

Rhizobial surface polysaccharides include capsular polysaccharides (CPS), exopolysaccharides (EPS), lipopolysaccharides (LPS), and β-1,2-glucans. They are key molecules for the establishment of the symbiosis [79,80] and may play other roles such as preventing plant-defense responses, or protection against oxidative stress or antimicrobial compounds [30,81,82].

CPS have been reported in rhizobia and agrobacteria [79,83]. In *Sinorhizobium*, CPS show structural analogies to group II K-antigens found in *Escherichia coli* and are, therefore, known as K polysaccharides (KPS) [79]. Although KPS have only been described in sinorhizobia, a first genome draft of PRF 81 revealed three genes for KPS biosynthesis [32]. Two chromosomally located gene regions, known as *rkp-1* and *rkp2*, as well as a pSymB located *rkp-3* region are responsible for KPS biosynthesis in *S. meliloti*[79]. Here we found a cluster of genes homologous to *rkp-1* in the chromosomes of CIAT 899 and PRF 81. Genes in this region seem to determine the biosynthesis (*rkpA*), modification and transport (*rkpGHIJ*) of a lipophilic molecule that may serve as a lipid carrier or anchor for KPS [84,85]. The only other Rhizobiaceae with an *rkp-1* region is the S4 strain of *Agrobacterium vitis*[35], although the production of KPS by this strain has not been investigated. The *rkpU* gene located upstream of *rkpA* in sinorhizobia and putatively involved in KPS export [86] is also conserved in CIAT 899, PRF 81 and S4. The sinorhizobial *rkp-2* region is composed of *lpsL* and *rkpK*, genes that are involved in the biosynthesis of nucleotide-sugar precursors for KPS (*rkpK*) and LPS (*lpsL* and *rkpK*) [87]. In CIAT 899 and PRF 81, both genes were chromosomal, although the gene order is reversed in relation to *S. meliloti*. Similar *rkp-2* regions are found in the genomes of other *Rhizobium* and *Agrobacterium* strains. In *R. leguminosarum* and *R. rhizogenes*, *rkpK* homologues have been implicated in the biosynthesis of CPS, EPS or LPS [88,89]. The sinorhizobial *rkp-3* region contains genes involved in KPS polymerization and export, and also strain-specific genes that determine its sugar composition [79]. No matches to *rkp-3* genes were found in CIAT 899 or PRF 81, indicating that the polysaccharide produced by these strains is different from that produced by sinorhizobia. A cluster of 19 genes located in the CIAT 899 megaplasmid encode functions putatively related to CPS biosynthesis. Recently, an orthologous syntenic cluster of *R. rhizogenes* K84 was postulated to be involved in the biosynthesis of a CPS different from the typical high molecular weight sinorhizobial KPS [89]. The putative K84 polysaccharide seems to be required for normal attachment and biofilm formation on an abiotic hydrophobic surface, but not on tomato roots [89].

The EPS secreted by CIAT 899 consists of an octasaccharide subunit made of glucose and galactose, decorated with acetyl and pyruvyl groups [90]. The CIAT 899 EPS is structurally similar to succinoglycan (EPS I) produced by *S. meliloti*[90]. The *exo* genes of CIAT 899 were organized in a large cluster located in the megaplasmid. A homologous *exo* cluster was located in a putative megaplasmid contig of PRF 81 with gene products displaying identities between 82% and 98% with those of CIAT 899. The *exo* gene clusters of these strains differed in that *exoH* has become a pseudogene in CIAT 899. In *S. meliloti*, ExoH is responsible for the addition of the succinyl group to EPS I [91]. This is consistent with the absence of succinyl groups in the CIAT 899 EPS [90], and indicates that PRF 81 produces succinylated EPS. Inoculation of alfalfa with a *S. meliloti exoH* mutant results in ineffective nodules without intracellular bacteria [91]. EPS is not required for nodulation of common bean by CIAT 899, but may contribute to competitiveness [92]. As succinylation of EPS is important for normal nodulation of some hosts, differences in host range between CIAT 899 and PRF 81 may be expected. The arrangements of *exo* genes in both strains were highly similar to that reported for *S. meliloti* 1021 [93], except for the absence of *exoI* and *exoT* in CIAT 899 and PRF 81. CDS with similarities to *exoI* and *exoT* of *S. meliloti* 1021 were found elsewhere in the genomes of both strains.

LPS represent the major component of the outer leaflet of the outer membrane of Gram-negative bacteria. LPS are composed of three structural domains, lipid A, core oligosaccharide and the O-antigen polysaccharide [94]. *Rhizobium* lipid A molecules typically contain a secondary very long acyl chain (VLAC) at the 2’ position [95]. This VLAC seems to confer stability to the membrane and is required for the establishment of a normal or fully effective symbiosis [96,97]. As in other rhizobia, lipid A of CIAT 899 harbors 27-hydroxyoctacosanoic acid as VLAC, however, it also contains 29-hydroxytriacontanoic acid [98]. The *acpXL* and *lpxXL* genes encoding the specialized acyl carrier protein and transferase proteins required for incorporation of VLAC into lipid A, as well as four other genes responsible for the biosynthesis of this substituent, were found in the chromosomes of CIAT 899 and PRF 81 arranged in a syntenic cluster that is also conserved in other rhizobia [96]. Putative LpxE and LpxF phosphatases, and an LpxQ oxidase were also found encoded in the chromosomes of CIAT 899 and PRF 81, suggesting that lipid-A molecules produced by these strains lack 1 and 4’ phosphate groups, and have a 2-amino-2-deoxy-gluconate moiety as described for the lipid As of *R. leguminosarum* and *R. etli*[95]. Dephosphorylation of lipid A in *R. etli* seems to confer resistance to antimicrobial peptides but is not required for symbiosis [82]. Glycosyltransferases, 76% identical to the core oligosaccharide mannosyltransferase LpcC from *R. leguminosarum*[99], were found encoded in the chromosomes of CIAT 899 and PRF 81. This is consistent with the observation that a mutation in *noeJ*, responsible for the biosynthesis of GDP-mannose, causes the production of LPS molecules with a truncated core in CIAT 899 [30]. No matches to other known genes involved in rhizobial core oligosaccharide biosynthesis were found in CIAT 899 and PRF 81, suggesting that sugar composition of their core oligosaccharides may be different from studied rhizobial strains. The O-antigen is the distal portion of the LPS molecule and can be highly variable from strain to strain [94]. In CIAT 899, the O-antigen units are composed of D-glucose, acetylated 6-deoxy-D-talose and L-fucose [100]. Two loci affecting O-antigen biosynthesis have been reported in CIAT 899 [30]. Both loci were mapped here in the chromosome. The first one includes a nucleotide sugar dehydratase gene, *lpsβ2*, putatively involved in UDP-D-QuiNAc biosynthesis, the donor of acetylquinovosamine that in other bacteria is the sugar linking the O-antigen and the core oligosaccharide [30]. An orthologue of *lpsβ2* was found in the chromosome of PRF 81. The second CIAT 899 locus contains the *wzmwzt* genes encoding an ABC type O-antigen transporter [30]. Interestingly, genome sequencing revealed that *wzmwzt* were part of a larger locus encompassing 22 genes predicted to be involved in polysaccharide biosynthesis. This gene cluster was located next to a tRNA-Gln gene, and contained the remnants of a *traG* gene and an IS transposase, suggesting that it was acquired by lateral gene transfer. Two genes in this cluster encoded a putative sugar transferase for the initiation of O-antigen biosynthesis and a putative WaaL O-antigen ligase. The WaaL protein of CIAT 899 showed hydropathy plots and conserved residues similar to characterized O-antigen ligases (Additional file 3A, B and C). To test the involvement of the CIAT 899 *waaL* gene in O-antigen biosynthesis, an insertional mutant was constructed. The mutant produced rough LPS molecules with normal migration behavior, indicating that lipid A and core oligosaccharide were not affected, but did not produce smooth LPS molecules (those carrying O-antigen), in agreement with the predicted function of WaaL as an O-antigen ligase (Additional file 3D). The mutant complemented *in trans* with a wild-type copy of *waaL* regained the ability to produce smooth LPS molecules (Additional file 3D). Interestingly, a putative O-antigen biosynthesis gene cluster was found in a similar location next to a tRNA-Gln gene in the PRF 81 chromosome. Homologues of *wzm* and *wzt*, as well as a putative *waaL* gene were found in this cluster, but the remaining genes were different from those of CIAT 899. This is consistent with previously reported LPS-profile differences between CIAT 899 and PRF 81 [15]. Although the exact role of O-antigen during rhizobium-legume interactions is unknown, abnormal symbioses are elicited by mutants lacking this LPS domain, with the effects being more drastic when a legume forming determinate nodules, such as common bean, is involved [79]. Recently, an intact O-antigen was shown to be required for normal endophytic colonization of maize by CIAT 899 and evidence for a possible role of LPS as a protectant against maize lipophilic antimicrobial compounds was presented [30].

### Secretion and plasmid-transfer systems

The conserved general secretion (*sec*) and twin-arginine translocation pathways responsible for the majority of protein export into the periplasm were identified in the CIAT 899 and PRF 81 genomes. Exoprotein secretion through the Gram-negative bacterial envelope is accomplished by several classes of transport system. Some of them such as the Type II and V systems depend on the activity of the *sec* pathway for delivery of their substrate proteins into the periplasm, whereas the remaining systems (type I, III, IV, VI, VII) are able to directly pick proteins from the cytosol [101]. Secreted proteins can fulfill general functions but also may be be specifically required for interaction with eukaryotic hosts or also with other bacteria [101]. In rhizobia, type I, III, IV and VI secretion systems have been shown to be involved in symbioses with legume hosts [4,102]. Only type I, IV and V systems were identified in the genomes of CIAT 899 and PRF 81.

Protein secretion by the type I secretion system (T1SS) occurs through an oligomeric protein channel composed of an inner membrane ATP-binding cassette (ABC) protein, a largely-periplasmic membrane fusion protein (MFP), and a pore-forming outer-membrane protein (OMP) [103]. The ABC- and MFP-encoding genes are usually arranged in an operon, whereas the OMP component may be encoded by a dedicated adjacent gene, or by *tolC* encoding a common OMP able to interact with several transporters [104]. T1SS protein substrates typically contain carboxy-terminal, glycine-and aspartate-rich repeats known as repeat-in-toxin (RTX) [105] and are often located close to ABC and MFP genes. Two and three putative T1SSs were identified in the genomes of CIAT 899 and PRF 81, respectively, all located in their megaplasmids. A T1SS shared by both strains was composed of an ABC-MFP gene operon followed with a divergently oriented gene encoding a protein 83% similar to the endoglycanase ExsH of *S. meliloti*. ExsH contributes to the production of low-molecular-weight EPSI, but is not involved in symbiosis [106]. The second T1SS of CIAT 899 comprised ABC and MFP genes and a putative T1SS substrate gene. The ABC and MFP components were highly similar (≥79%) to RspD and RspE required for rhizobiocin secretion in *R. leguminosarum*[107]; nevertheless, the CIAT 899 T1SS substrate protein was unrelated to known bacteriocins and did not show any conserved domain, besides RTX. The second and third T1SS of PRF 81 were composed of ABC-MFP and OMP-ABC-MFP genes, respectively, encoding proteins without close homologues in the databases, and did not have nearby genes encoding T1SS substrate proteins. The only additional identifiable T1SS substrate of PRF 81 possessed copies of the VCBS-repeat domain, suggesting that this protein acts as an adhesin. Other known rhizobial T1SS-exported proteins that do not possess the typical RTX motif, like the glycanases PlyA, PlyC and Egl, and the rhizobial adhering (Rap) proteins, did not have counterparts in the genomes of CIAT 899 or PRF 81.

The type V secretion system (T5SS) allows secretion of large proteins that act as virulence factors [108]. Three subclasses of T5SS are recognized. The autotransporter (AT) subclass consists of multidomain proteins, including a passenger domain that is the functional secreted moiety, and a pore-forming β-barrel domain that mediates secretion through the outer membrane. The trimeric autotransporters (TAA) subclass proteins contain only one third of the β-barrel domain, so they must form trimers in order to be secreted. In the two-partner system (TPS) subclass, the passenger and β-barrel domains are encoded in distinct polypeptides called TpsA and TpsB, respectively [108]. The genomes of CIAT 899 and PRF 81 each encoded two TPS and one TAA systems. The TPS systems of both strains were related, as each TpsB protein has a close homologue (>80% identical) encoded in the other strain, but the cognate TpsA secreted proteins have diverged (<60% identity). TpsA proteins of both strains were putative filamentous hemagglutinins (FHA). FHA act as adhesins and are virulence factors of animal [109] and plant [110] pathogens, but a possible role in symbiotic relationships has not been determined. The second subclass of T5SS systems found in the CIAT 899 and PRF 81 genomes encoded TAA proteins 69% identical to each other and which were similar to YadA-like adhesins. YadA is a virulence factor of *Yersinia enterocolitica* that mediates autoagglutination, adhesion to host cells and also protects the bacterium against complement and defensin lysis [111]. YadA-like adhesins have not been characterized in the context of plant-microbe interactions.

Type IV secretion systems (T4SS) are able to transfer protein or nucleoprotein complexes across membranes [112]. We found T4SSs in two of the CIAT 899 plasmids and in all four PRF 81 plasmids. Two P-type T4SS were identified in PRF 81, one located in pPRF81a and the other in the megaplasmid. The T4SS located in pPRF81a was composed of *virB/virD4* genes and is most similar to the T4SS of plasmid pAtK84c of *R. rhizogenes* K84 [113]. The *virB*/*virD4* system of *Agrobacterium tumefaciens* is responsible for transfer of tumorigenic DNA (T-DNA) into plant cells [114], while in *M. loti* R7A, a VirB/VirD4 system acts in the translocation of effector proteins into host cells, affecting the symbiosis in a host-dependant manner [115]. We did not find homologues to the two-component regulatory system VirA/VirG that controls expression of *virB*/*virD4* systems in *A. tumefacciens* and *M. loti*, nor to *M. loti* T4SS effector proteins [115,116]. In contrast, a *traA* gene encoding a relaxase/nuclease, a component of the DNA transfer and replication (Dtr) system that recognizes and cleaves at the origin of transfer during conjugation, was found 10.4 kb downstream of *virD4*, indicating that the T4SS of pPRF81a is involved in plasmid conjugation rather than protein secretion. The P-type T4SS of the PRF 81 megaplasmid was similar to conjugation machineries present in *R. etli* CFN42 pSym, *S. meliloti* 1021 pSymA, and *A. tumefaciens* C58 pAt plasmid [117], and included the regulatory gene *rctA* that is required for repression of conjugative transfer [118]. A *traAtraCDG* Dtr system was located 13.4 kb away from the PRF 81 megaplasmid *rctA-virB* cluster, nevertheless, the *traA* gene is truncated suggesting that this conjugation system may not be functional.

F-type *tra*/*trb* T4SSs were identified in the tropici pSym, in pRtrCIAT899a, and in pPRF81b. Sequence analysis of individual *tra*/*trb* genes indicated that these T4SS were closely related to various conjugation systems, including those present in *R. etli* CFN42 p42a and *S. fredii* GR64 p64a plasmids [119]. pRtrCIAT899a is self-transmissible (our own unpublished data), whereas pPRF81b may have lost this ability as it lacks *traCDG* genes. All F-type T4SSs identified in the CIAT 899 and PRF 81 genomes were adjacent to *repABC* genes, and included *traI*, *traR* and *traM* genes, indicating that they are regulated by quorum-sensing mechanisms involving *N*-acyl homoserine lactones [119]. Interestingly, an IS*256* inserted upstream of *traR* in the CIAT 899 pSym may have disrupted its promoter leading to a constitutive repression of the conjugation system of this plasmid.

### Iron uptake

Iron is an essential nutrient that is not readily available under normal conditions, because it is present mostly as insoluble forms. Thus, the efficient acquisition of this element may improve bacterial survival and confer competitiveness [120]. Under Fe-replete conditions, bacteria repress the expression of Fe-uptake systems through special transcriptional regulators. CIAT 899 and PRF 81 have genes for RirA that has replaced Fur as the master regulator of iron-responsive genes in the Rhizobiaceae; and Irr, a second, minor-acting Fe-responsive regulator present only in the Rhizobiales and Rhodobacterales [121]. Both strains also possess a *fur* homologue. In rhizobia, *fur* gene products do not regulate Fe-uptake systems; instead they act as regulators of Mn transporters and the genes have been renamed as *mur*[121].

To capture iron, bacteria and fungi can produce siderophores, high-affinity low-molecular-weight ligands. CIAT 899 possesses a hydroxamate siderophore-biosynthesis gene cluster, some genes of which are similar to the vicibactin *vbs* genes of *R. leguminosarum*[122]. No genes for siderophore synthesis were identified in PRF 81, suggesting that this strain is adapted to obtain chelated iron forms from external sources, a strategy used by the endophytic bacterium *Azoarcus* sp. BH72 [123]. External siderophore-iron complexes are recognized and translocated to the periplasm by outer membrane TonB-dependent receptors, and once in the periplasm they are internalized into the cytoplasm by ABC-type transporters. Bacteria not producing siderophores can utilize “xenosiderophores” by making appropriate receptors and transporters. We found three and one siderophore-receptor genes in CIAT 899 and PRF 81, respectively, and five ABC transporters of the siderophore type encoded in each strain genome. A CIAT 899 receptor/transporter pair similar to several ferric hydroxamate utilization (Fhu) systems was encoded near the siderophore-biosynthesis genes. The second receptor of CIAT 899 was located adjacent to one siderophore transporter and was 55% similar to the anguibactin catechol-siderophore receptor FatA from *Vibrio anguillarum*[124]. The third CIAT 899 receptor was 85% similar to the single PRF 81 siderophore receptor, and both were 59–62% similar to ShmR of *S. meliloti*, a receptor required for heme utilization [125]. CIAT 899 and PRF 81 shared a transporter highly similar to the Hmu system of *R. leguminosarum* that is involved in heme utilization as an iron source [126].

We found three ABC transporters of the ferric ion (Fe^+3^) type in the genomes of both strains. Fe^+3^ may be more available in tropical acid soils where CIAT 899 and PRF 81 evolved than in neutral soils, thus explaining the presence of these transporters. Citrate can act as a weak siderophore and may capture enough iron to sustain growth in acid soils [127]. CIAT 899 and PRF 81 possess a citrate synthase gene in their pSyms. We have observed that the citrate synthase gene located in the tropici pSym is induced under low-iron conditions [128], suggesting that both strains do, indeed, use citrate as a siderophore. In contrast to other siderophores, ferric citrate utilization in rhizobia does not seem to require a TonB-dependent receptor [126]. CIAT 899 and PRF 81 have a gene encoding a bacterioferritin used for intracellular iron storage.

### Phytohormone production

Several plant hormones, such as ethylene, auxins, gibberellins, have been reported to influence root colonization, and distinct stages of nodule development [129]. We found genes involved in plant-hormone metabolism in CIAT 899 and PRF 81 genomes. An *acdS* gene, with high identity (~84%) to homologues located in symbiosis islands of mesorhizobial strains R7A and MAFF303099, was found in the tropici pSym (Figure 3B). *acdS* encodes 1-aminocyclopropane-1-carboxylate (ACC) deaminase which degrades ACC, the immediate precursor of ethylene in higher plants [130]. Ethylene inhibits rhizobial infection [129] and it has been shown that strains engineered to overexpress ACC deaminase activity have enhanced symbiotic proficiency [131,132]. The *acdS* gene of *R. leguminosarum* strain 128C53K is regulated by a leucine-responsive regulatory protein that is encoded by the *lrpL* (*acdR*) gene positioned upstream of *acdS*[133]. In the vicinity of the tropici pSym *acdS*, a gene encoding a two-component transcriptional regulator was found that may have a role in its regulation.

Many plant-associated bacteria, including rhizobia, synthesize auxins, in particular indole-3-acetic acid (IAA) [134]. Genomic analysis suggests that IAA biosynthesis may proceed through two and three tryptophan-dependant pathways in CIAT 899 and PRF 81, respectively. In the indoleacetamide pathway, tryptophan is converted to IAA in two consecutive reactions catalyzed by tryptophan monooxygenase (IaaM) and indoleacetamide hydrolase (IaaH). A putative monooxygenase gene, distantly related to IaaM from *A. vitis*[135] was found coded in the tropici pSym. An *iaaH* gene encoding a protein 47% similar to IaaH from *A. vitis* could be found only in the PRF 81 megaplasmid, indicating that the indoleacetamide pathway is operative in this strain but not in CIAT 899. In a second pathway, indole-3-acetonitrile can be directly converted to IAA by a nitrilase, or by the action of a nitrile hydratase it can be converted to indoleacetamide that is then metabolized by IaaH. Genes encoding proteins with similarities to nitrilase and two subunits of a nitrile hydratase were found in the chromosomes of CIAT 899 and PRF 81 strains. The third IAA biosynthesis route, the indolepyruvate pathway, requires tryptophan transferase and indole-3-acetaldehyde oxidase proteins. Homologues to *Sinorhizobium* sp. NGR234 genes encoding those proteins were located in the tropici pSym (Figure 3B). The NGR234 genes are flavonoid-inducible although they are not required for nodulation [136]. Interestingly, the transcriptional regulator *nodD5* gene is located close to the IAA genes in the tropici pSym, suggesting that it may regulate their transcription in response to plant flavonoids. To test this hypothesis, IAA production was quantified in *R. leucaenae* CFN 299 and in its 200-kb pSym deleted mutant CFN 299–10 when exposed to various flavonoids. We observed that IAA production was enhanced by naringenin, genistein, luteolin, chysin and apigenin in CFN 299, but not in CFN 299–10 (Additional file 4), clearly indicating that a flavonoid-inducible IAA biosynthesis system was coded in the tropici pSym.

An operon involved in gibberellin biosynthesis has been identified in the *B. japonicum* genome [137]. The tropici pSym possessed a cluster of genes encoding three putative cytochrome P450s, a sterol dehydrogenase-like enzyme, a geranylgeranyl diphosphate synthase, an ent-copalyl diphosphate synthase, and an ent-kaurene synthetase showing >87% identity to the *B. japonicum* homologues. We observed that similar gene clusters are present in the symbiosis island of *M. loti* and in the pSyms of *Sinorhizobium* sp. NGR 234 and *R. etli* CFN 42.

### Resistance to antimicrobials

One distinguishing phenotype of *R. tropici* CIAT 899 is its high resistance to several antimicrobial compounds, including antibiotics and pesticides, in comparison to other rhizobia nodulating common bean, such as *R. etli* and *R. leucaenae*[14,28]. PRF 81 is as resistant as CIAT 899 to many of these antibiotics (Additional file 2). CIAT 899 also shows high resistance to other types of antimicrobial compounds, such as the fungicides Thiram and Captan that diminish the survival of rhizobia in seed-applied inoculants [29,30]. Resistance to antimicrobial compounds can be conferred by the action of efflux pumps that transport those compounds outside the cells [138]. We performed a systematic search for genes encoding drug-efflux pumps in the genomes of CIAT 899, PRF 81, other rhizobia, and in the genome of *Burkholderia cenocepacia* J2315. This latter strain was included as an example of a multidrug-resistant bacterium [139]. Interestingly, the genomes of CIAT 899 and PRF 81 had more genes encoding putative efflux pumps than the majority of other rhizobia (Table 2). *B. japonicum* USDA 110 was the only rhizobial strain with a similar number of genes, although when normalized against genome size, CIAT 899 and PRF 81 genomes showed the highest density of these transporters per Mb. Similarly, *B. cenocepacia* had more genes than CIAT 899 and PRF 81, but not necessarily at higher density (Table 2). The abundance of efflux pumps in their genomes may explain the higher resistance of CIAT 899 and PRF 81 to many antimicrobials in comparison to other rhizobia.

**Table 2:** Number of genes encoding antimicrobial efflux pumps in the genomes of rhizobia

	**Number of transporters**									
**Superfamily/Family**	**Rt**^**a**^**CIAT899**	**Rsp PRF81**	**Re CFN42**	**Rl 3841**	**Sm 1021**	**Ssp NGR234**	**Mh MAFF**	**Bj USDA110**	**Ac ORS571**	**Bc**^**b**^**J2315**
MFS ^c^	46	43	31	36	32	24	35	34	21	53
RND ^c^	12	11	8	8	8	10	10	19	10	14
MATE	4	4	3	4	2	2	4	7	4	3
SMR ^c^	2	2	2	2	4	5	2	3	2	2
Total number	64	60	44	50	46	41	51	63	37	72
Number/Mb	9.6	8.4	6.8	6.4	6.8	5.9	6.7	6.9	6.9	9.3

^a^ Rt, *R. tropici*; Rsp, *Rhizobium* sp.; Re, *R. etli*; Rl, *R. leguminosaum;* Sm, *S. meliloti*; Ssp, *Sinorhizobium* sp.; Mh, *M. huakuii*; Bj, *B. japonicum*; Ac, *A. caulinodans*; Bc, *Burkholderia cenocepacia.*

^b^*Burkholderia cenocepacia* J2315 was included as an example of a multiresistant bacteria.

^c^ Only members belonging to antimicrobial extrusion families were counted. For multigene systems, only the inner membrane component was counted.

The activity of enzymes that modify or break down antibiotics represents another mechanism for resistance [140]. We found 15 and 17 genes encoding proteins of the β-lactamase family in the genomes of CIAT 899 and PRF 81, respectively. Some of them may explain the resistance of both strains to carbenicillin. Both genomes also encoded one or two aminoglycoside phosphotransferases, aminoglycoside acetyltransferases, and streptomycin kinases. Additionally, the presence of one and two genes for putative chloramphenicol acetyltransferases in CIAT 899 and PRF 81, respectively, may contribute to their known resistance to chloramphenicol (Additional file 2).

Soil can be an inimical environment and the production of antimicrobials represents a strategy for clearing niches of competing microorganisms or limiting their growth [141]. In this context, intrinsic resistance to antimicrobials may be advantageous for survival and thriving in the soil [142]. Bacteria colonizing plant tissues are also challenged with a wide range of antimicrobials produced by plants as defense mechanisms [143]. Even mutualistic symbionts like rhizobia are exposed to these compounds when interacting with their legume partners [144,145], and studies suggest that efflux pumps can protect rhizobia and thus promote effective nodulation of some hosts [146,147]. Therefore, the large number of efflux pumps of CIAT 899 and PRF 81 may contribute to the broad host ranges of these bacteria by allowing them to repel the antimicrobial compounds produced by legumes.

### Other genes involved in competitiveness

Pili or fimbriae are proteinaceous, filamentous surface appendages that are involved in bacterial surface attachment and virulence [148]. CIAT 899 and PRF 81 genomes possessed a cluster of genes for pili assembly through the chaperone/usher pathway. Several rhizobia produce pili [149] that are involved in root attachment and nodulation [150]. Both strain genomes also harbored a gene cluster for the biosynthesis of Type IV pili. These pili are involved in host-cell attachment, biofilm formation, and also in twitching motility due to their capacity to retract [148]. Type IV pili have not been studied in rhizobia although they are involved in plant colonization in the N_2_-fixing bacterium *Azoarcus* sp. [151]. Although twitching motility has not been reported in CIAT 899 or PRF 81, both strains display swimming motility, a characteristic that may contribute to efficient host colonization [152]. A flagellum-biosynthesis gene cluster adjacent to a chemotaxis gene cluster was encoded in both strain genomes.

Opines are compounds produced by plant tumors or hairy roots induced by pathogenic *Agrobacterium* species. The genes required for opine synthesis are contained in the T-DNA that agrobacteria transfer to the plant cells. Opines serve as carbon and nitrogen sources for the bacteria. In a survey of *Agrobacterium* and rhizobial species, it was reported that rhizobia—with the possible exception of nopaline by *B. japonicum*—were unable to use opine or nopaline as carbon and nitrogen sources [153]. Interestingly, we identified genes for the uptake of octopines (*occMPQT*) in CIAT 899 and PRF 81 genomes, and nopaline (*nocTMQ*) in PRF 81. We have previously observed that CIAT 899 is able to use octopine as a source of carbon and nitrogen (E. Martínez-Romero, unpublished data). This unusual characteristic of CIAT 899 and PRF 81 may be used in conjunction with plants engineered to produce opines as an strategy to improve competiveness of inoculant strains against indigenous rhizobial populations [154].

The ability to catabolize root-exudate sugars has also been shown to be an advantageous trait for root colonization or nodulation. CIAT 899 and PRF 81 megaplasmids encoded an orthologue of the rhamnose-uptake and catabolism locus described in *R. leguminosaum* and required for competitive nodulation of clover (*Trifolium repens*) [155]. Likewise, CIAT 899 and PRF 81 chromosomes included orthologues of the *iolDEB* genes involved in inositol catabolism and nodulation in *R. leguminosarum*[156]. Both strain genomes carry two inositol ABC transporters, one in the megaplasmid and another in the chromosome. It was previously shown that CIAT 899 carry *teu* genes in pRtrCIAT899a which encode a putative sugar-uptake ABC transporter that is induced by root exudates of common bean and *M. atropurpureum*, and that is required for nodulation competitiveness [157]. We now report that the *teu* locus is present in the pPRF81b plasmid.

An orthologue of the transcriptional regulator RosR of *R. etli* CFN 42 was found encoded in the chromosomes of both strains. RosR belongs to a family of transcriptional regulators like MucR of *S. meliloti* and Ros of *Agrobacterium* species that are conserved in the Rhizobiaceae and that influence the expression of genes involved in nodulation or virulence [158]. CIAT 899 and PRF 81 genomes coded for eight homologues of the *Bartonella bacilliformis ialB* gene. IalB is required for erythrocyte adherence and invasion by bartonellas [159]. Two loci required for cellulose synthesis found in each genome may be involved in root attachment through cellulose fibrils as in *R. leguminosarum*[160]. A cellulase, 70% identical to CelC2 of *R. leguminosarum* ANU843, and an endoglucanase, 40% identical to EglA of *Azoarcus* sp. BH72, coded in both strain genomes may promote host-cell entry and dissemination by localized cell-wall degradation [161,162]. Vitamin prototrophy may confer an advantage for competitive rhizosphere colonization [163]. Except for biotin, CIAT 899 and PRF 81 harbor similar complements of genes required for vitamin biosynthesis. CIAT 899 lacks biotin-biosynthesis genes, whereas PRF 81 harbored *bioBFDAZ* genes in its megaplasmid. Both strains may use external biotin through the action of transport systems coded by *bioMN* and *bioY*. Although CIAT 899 appears to be a biotin auxotroph, it may be highly efficient at scavenging biotin as it possesses an additional BioY transporter.

### pH stress

CIAT 899 and PRF 81, considered acid-resistant strains [13,15], may face low-pH stress in acid soils and also in the rhizosphere and inside the symbiosome. Rhizobial mechanisms involved in survival and growth under acidic conditions are not well understood. In CIAT 899, a locus including the low pH inducible *lpiA* and the acid tolerance and virulence *atvA* genes is up-regulated by acidic pH [27]. This locus was found to be conserved in PRF 81 and in many acid-tolerant, as well as acid-sensitive rhizobia and agrobacteria. *lpiA* is required for the synthesis of lysyl-phosphatidylglycerol, which confers resistance to antimicrobial peptides and is required for nodulation competitiveness, although not for acid resistance [164]. AtvA and its orthologue AcvB of *A. tumefaciens* are required for acid tolerance [27], but their function is unknown. Based on similarities to lipases, it has been proposed that AtvA/AcvB is involved in membrane lipid metabolism required for adaptation to acid conditions [27]. Indeed, cell-envelope modifications are common in bacteria exposed to acid stress [165-167]. In CIAT 899, acidity induces an increase in the amount of ornithine lipids (OL) in the membrane and the presence of a hydroxylated OL species produced by OlsC seems to be important for acid resistance [26]. Some Proteobacteria, including PRF 81, carry *olsC* genes and their presence may correlate with increased tolerance to acidic conditions [26,27]. An *eptA* homologue encoding a putative lipid A phosphoethanolamine transferase was found in CIAT 899 and PRF 81. In *E. coli* and *Salmonella typhimurium*, *eptA* is induced under mildly acidic conditions and it has been shown that *eptA* confers acid resistance in *Shigella flexneri* 2a [168]. *eptA* homologues are present in *R. rhizogenes* K84, agrobacteria and sinorhizobia, but not in other *Rhizobium* species. Cyclopropane-containing fatty acids are thought to improve acid tolerance by reducing membrane permeability to H^+^[169]. CIAT 899 and PRF 81 chromosomes possessed two genes encoding cyclopropane-fatty-acyl-phospholipid synthases (*cfa*). In addition, CIAT 899 possessed three *cfa* genes, one in its megaplasmid and two in pRtrCIAT899a. EPS production has been correlated with acid tolerance and *exo* genes are among the most induced in *S. meliloti* and *A. tumefaciens* after an acid shock [170-172]. CIAT 899 and PRF 81 produce copious amounts of EPS, a characteristic that may contribute to their resistance to acid.

Under acidic stress, some bacteria can reverse membrane potential—normally negative inside and positive outside—by accumulating positively charged molecules, such as potassium ion (K^+^), in order to repel protons and slow down H^+^ influx [173]. The CIAT 899 and PRF 81 genomes encoded a homologue of *kcsA*, a *Streptomyces lividans* ion channel that opens at low pH allowing K^+^ entrance into the cell. Riccillo *et al.*[24] found that the glutathione synthase *gshB* gene is required for CIAT 899 tolerance to acidic stress, its activation occurring under low-pH conditions [174]. The inability of a CIAT 899 *gshB* mutant to grow in low-pH media was related to a diminished capacity to accumulate K^+^ at low pH, and it was proposed that a KefB/KefC glutathione-regulated K^+^ efflux transporter may be too active in the absence of glutathione [24]. In support of that hypothesis, we found a putative *kefB*/*kefC* homologue in the genome of CIAT 899 and a highly similar gene in the PRF 81 genome.

Prokaryotic ClC-type chloride channels function as H^+^/Cl^–^ antiporters, able to extrude H^+^ under low-pH conditions [175]. Interestingly, we found four chromosomal genes for this type of antiporters in CIAT 899 and PRF 81. These paralogous genes probably make different contributions to pH homeostasis as a CIAT 899 mutant in one channel (*sycA*) was apparently not impaired in growth at pH 4.5, but rather showed symbiotic defects [25].

Enterobacteria possess efficient systems to cope with extremely acidic conditions that rely on H^+^ consumption by decarboxylation of externally-supplied amino acids and on the action of antiporters that expel the decarboxylated products in exchange for new amino acid substrates [173]. CIAT 899 and PRF 81 seem to lack these systems as we did not find orthologues to the amino acid decarboxylase or antiporter genes used by enterobacteria.

pH homeostasis in alkaline conditions relies on the action of antiporters that direct proton influx to reduce internal pH in exchange for the monovalent cations Na^+^ or K^+^, depending on their availability [173]. Homologues of the multicomponent *pha1*[176] and *pha2*[177] cation/H^+^ antiporter systems described in sinorhizobia for alkaline pH adaptation were found in CIAT 899 and PRF 81. The CIAT 899/PRF 81 *pha2* system may also be involved in the tolerance of these strains to high NaCl concentrations, as observed in sinorhizobia [177]. Both strain genomes carried additional cation/H^+^ antiporters of the monocomponent type (two in CIAT 899 and one in PRF 81) that may contribute to pH homeostasis or resistance to strongly saline conditions.

### Temperature stress

CIAT 899 as well as PRF 81 can grow at temperatures up to 40°C and this characteristic is thought to be important for their success as tropical inoculant strains [13,15]. Several proteins, collectively known as heat-shock proteins (HSPs), are induced after exposure to high temperatures. In *E. coli*, transcription of many genes encoding HSPs is under the control of the alternative sigma factor RpoH. Proteobacterial species commonly have more than one *rpoH* homologue, such as two in *R. etli*, *S. meliloti* and *Brucella melitensis*, and three in *B. japonicum*. Usually, one of the *rpoH* genes is mainly responsible for coping with heat shock, whereas the others may complement the response or be involved in responses to other stresses. CIAT 899 and PRF 81 possessed two chromosomal *rpoH* genes, one of which is a close relative to *rpoH1* of *R. etli* and *S. meliloti*, and to *rpoH2* of *B. melitensis*, responsible for heat-shock responses in those bacteria. In many bacteria, like *B. japonicum*, the positive control of the heat-shock response by RpoH is combined with a negative control by the HrcA repressor [178]. One putative *hrcA* gene was found in CIAT 899 and PRF 81, although it may have a restricted role, as has been shown for *hrcA* in *A. tumefaciens*[179]. Common HSPs involved in protein folding such as DnaK-DnaJ-GrpE composing the DnaK chaperone system, and GroEL-GroES composing the GroE chaperonin machinery were found encoded in the chromosomes of CIAT 899 and PRF 81, whereas the HtpG chaperone is encoded in their megaplasmids. In contrast with other rhizobia, a single *groELgroES* operon was found in CIAT 899 and PRF 81. Both strains possessed several homologues of the *ibpA*/*B* genes encoding small HSPs of the HSP20 family involved in reversing protein aggregation induced at high temperatures [180]. Interestingly, two of the small HSPs genes resided in the tropici symbiotic plasmid, suggesting a possible role during symbiosis. Four homologues of the high temperature requirement HtrA protease/chaperone—required for degradation of misfolded and mislocalized cell-envelope proteins [181]—were encoded in both strains. Several *htrA* homologues are also present in the genomes of other bacteria [181]. In *S. meliloti* and *Brucella abortus*, mutations in one *htrA* paralogue have only a small effect on growth at high temperatures [182,183], probably due to functional redundancy. Other common components of the heat-shock response such as the Clp and Lon protease systems, and the translation elongation factor LepA were also found in the CIAT 899 and PRF 81 genomes. The differential expression of several PRF 81 proteins under high-temperature stress such as DnaK, GroEL, and the translation factors EF-Tu, Ef-G and IF2, was recently demonstrated [184]. It is noteworthy that some HSPs also play protective roles during other stressful conditions like the DnaK machinery during hyperosmotic salt stress [185] and HtrA in oxidative damage [183].

As observed during heat shock, cells respond to cold shock by producing proteins known as cold-shock proteins (CSPs) to overcome the deleterious effects of low temperatures [186,187]. Homologues to the CSPs-coding genes *cspA*, *cspB*, *cspC* and *cspG* were present in duplicate in CIAT 899 and PRF 81 except for *cspB*, with only one copy in both strains, and *cspC*, of which there was only one copy in PRF 81. CspA, the major CSP in *E. coli*, is an RNA chaperone thought to facilitate translation by destabilizing mRNA secondary structures formed at low temperatures. CspA and the homologous RNA chaperones CspE and CspC are also transcription antiterminators [186]. Although the transcriptional activation of a *cspA* homologue in *S. meliloti* upon cold shock has been reported [187], its involvement, or of any other rhizobial CSP gene, in growth at low temperatures has not been documented.

### Osmotolerance

Bacteria exposed to osmotic stress react with mechanisms to avoid leakage of intracellular water in hypertonic environments, such as those with high concentration of salts; or to prevent cell burst due to excessive water influx in hypotonic environments [188]. K^+^ uptake after an osmotic upshift is a rapid response to preserve cell turgor [189]. CIAT 899 and PRF 81 genomes encoded a chromosomally-located K^+^-uptake protein (*kup*) gene specifically required for growth under highly osmotic conditions [185,189]. An additional *kup* gene, found in the PRF 81 megaplasmid, was similar to uncharacterized homologues harbored by *Bradyrhizobium* and *Methylobacterium*. The conserved KdpABC K^+^-transporting ATPase required for osmoadaptation to moderate concentrations of ionic solutes and to normal K^+^ homeostasis was found encoded in the megaplasmids of both strains [189]. Trk, the main system involved in K^+^ accumulation after an osmotic upshift in *S. meliloti*[189], was not found in the genome of either strain.

Biosynthesis and intracellular accumulation of molecules known as compatible solutes, such as trehalose, glycine betaine, proline betaine or ectoine, are common responses under high osmolarity stress conditions [190]. Exogenous compounds like choline or choline sulfate can be taken up by the cell and act as osmoprotectants or induce the biosynthesis of compatible solutes [190]. Trehalose accumulation is osmoregulated in CIAT 899, and growth of this strain in a high salt medium is improved when glycine betaine or choline is present [191,192]. Trehalose biosynthesis in rhizobia not only confers osmotolerance, but is also involved in nodulation competitiveness [193-195]. Chromosomes of CIAT 899 and PRF 81 encoded the enzymes OtsA and OtsB for trehalose biosynthesis from UDP-glucose and glucose 6-phosphate [194]. CIAT 899 possessed an additional pathway involving trehalose synthase, TreS, which converts maltose into trehalose [193]. Biosynthesis of glycine betaine from choline may proceed in both strains by the activities of choline dehydrogenase BetA and betaine aldehyde dehydrogenase, BetB, that were encoded in their chromosomes in close vicinity to their regulatory protein BetI [196]. Choline sulfate may also be used as a precursor of glycine betaine biosynthesis by the action of choline sulfatase BetC, which was encoded elsewhere in the chromosomes of both strains in contrast to *S. meliloti* where it is linked to *betAB*[196]. Both strains could take up external choline by a homologue of the high-affinity ChoXWV ABC-type transporter [197] also encoded in their chromosomes. ChoXWV belong to the QAT family that includes importers for glycine betaine, proline betaine, proline and histidine. CIAT 899 and PRF 81 encoded three and four other QAT transporters, respectively. The activity of QAT systems may be required for catabolism of their transported substrates rather than for osmotolerance [197], nevertheless, at least one of the additional QAT systems encoded in each genome had an ATPase component with CBS domains whose presence has been correlated with osmoregulatory functions [198]. CIAT 899 and PRF 81 megaplasmids each encoded one orthologue of the *S. meliloti* PrbABCD transporter for uptake of proline betaine at low and high osmolarities [199]. A putative diaminobutyrate-pyruvate aminotransferase *ectB* gene was found in CIAT 899 and PRF 81, but not the genes required for the remaining steps of ectoine biosynthesis from L-2,4-diaminobutyrate [200]. We did not find genes related to the biosynthesis of the dipeptide N-acetylglutaminylglutamine amide identified in osmotically stressed cultures of *S. meliloti*[201].

Increase in cell turgor under hypo-osmotic conditions is counteracted by the release of internal solutes, mainly K^+^, through mechanosensitive channels that respond to stretching of the cell membrane [202]. The CIAT 899 and PRF 81 genomes encoded for a large conductance mechanosensitive channel MscL, and five and four small conductance mechanosensitive channels MscS, respectively. Expression of two MscS genes in *R. leguminosarum* has been shown to be upregulated in the rhizosphere [203], probably indicating that rhizobia face osmotic stress in this environment. Periplasmic glucans (PGs) are polysaccharides composed of glucopyranosyl residues linked with β-glycosidic bonds that accumulate in the periplasm especially under low osmotic conditions, and are thus known as osmoregulated PGs [204]. PGs are required for growth of *Agrobacterium* and *Sinorhizobium* in hypo-osmotic media and for proper interaction with their plant hosts [204]. Genes *ndvB* and *ndvA* encoding the cyclic β-1,2-glucan synthase and exporter, respectively, were found in CIAT 899 and PRF 81. Interestingly, while NdvA exporters of both strains were 97% identical, the NdvB synthases showed an identity of only 67%. The NdvB synthase of CIAT 899 was similar to homologues present in other rhizobia and this strain produces a cyclic (1 → 2)-β-glucan PG formed by 17 β-glucopyranose units that is structurally similar to PGs produced by other Rhizobiaceae [191,205]. In contrast, the PRF 81 *ndvB* gene is divergent in comparison to those of other Rhizobiaceae and is not linked to *ndvA* as in other rhizobia, indicating that this strain produces structurally dissimilar PGs.

Aquaporins are water-selective channel proteins that mediate the influx and efflux of water to and from the cell in response to changes in osmolarity. Aquaporins are widely distributed in animals and plants, but are less prevalent in bacteria [206]. A role of bacterial aquaporins in osmoadaptation has been suggested based on studies with *E. coli* and *B. abortus*[206,207]. Two aquaporin genes were found in each CIAT 899 and PRF 81 genome, one in the chromosome and the other in the pSym. The chromosomal aquaporin genes of both strains were 97% identical to each other, and displayed 73–74% identity to the *B. abortus* aquaporin, which is required for prolonged growth in media of low osmolarity [207]. The pSym aquaporin genes were only 42–43% identical to the chromosomal genes, but 76% identical to the sole aquaporin gene of *R. etli* CFN 42. The CFN42 gene is located in its pSym and has been shown to be upregulated in bacteroids [208] suggesting a role in symbiosis.

### Oxidative stress

Oxidative stress occurs when the cell cannot properly detoxify reactive oxygen species (ROS) or repair the damage they cause on cellular components like proteins, lipids and DNA. Bacteria like rhizobia are exposed to endogenous ROS, like superoxide anion, hydrogen peroxide (H_2_O_2_) and organic peroxides, as a result of their own normal metabolism, and to external ROS during interaction with other microorganisms or eukaryotic hosts. The regulators of oxidative stress response to H_2_O_2_ and superoxide, OxyR and SoxR, respectively, were found in CIAT 899 and PRF 81. *oxyR* was located in their megaplasmids, whereas *soxR* was located in their chromosomes. Two classes of superoxide dismutase (SOD) enzymes that convert superoxide into H_2_O_2_ and oxygen were found coded in the CIAT 899 and PRF 81 genomes. One was a Cu-Zn-SOD (SodC) and the other a Mn/Fe-SOD (SodM). Both genomes encoded a single catalase, 74% identical to KatG of *R. etli* CFN 42, a dual-function catalase/peroxidase responsible for decomposition of H_2_O_2_ and other peroxides, which is not required for nodulation or nitrogen fixation [209]. As in *R. etli*[209], the *katG* gene of CIAT 899 and PRF 81 was plasmid-located (megaplasmids), adjacent to *oxyR*.

A wide range of other enzymes with peroxidase activity and protective roles were coded in both genomes. Four and three organic hydroperoxide resistance (*ohr*) protein paralogous genes were found in CIAT 899 and PRF 81, respectively. As their name indicates Ohr proteins show a marked preference for organic peroxides as substrates. One of the *ohr* genes of CIAT 899 and PRF 81 was adjacent to its putative *ohrR* transcriptional regulator. Orthologues of this *ohr*/*ohrR* gene pair in *S. meliloti* and *A. tumefaciens* are required for resistance to organic hydroperoxide stress [210,211]. We identified four genes for non-heme chloroperoxidases in CIAT 899 and two in PRF 81. This class of enzymes catalyzes concomitant chlorination and peroxidation reactions and is involved in oxidative stress responses [212] and secondary metabolism [213]. Genes for peroxiredoxins of the alkyl hydroperoxide reductase (AhpC)/thiol specific antioxidant (TSA) family were also found in CIAT 899 and PRF 81, with one representative in the former and three genes in the latter. An AhpC/TSA gene has been shown to be upregulated after H_2_O_2_ exposure in *S. meliloti*[212]. CIAT 899 and PRF 81 also encoded one homologue of the bacterioferritin comigratory protein (BCP)-type of peroxiredoxins. AhpC/TSA and BCP peroxiredoxins play protective roles against oxidative damage, including lipid peroxidation [214]. Two genes encoding atypical-2-Cys peroxiredoxins were found in each genome, one in the chromosome and the other in the pSym. The latter gene was 80% identical to *prxS* of *R. etli* CFN 42, a peroxiredoxin located in the phaseoli pSym that is involved in bacteroid defence against oxidative stress [215]. A gene in each strain encoded a di-heme cytochrome c peroxidase, a periplasmatic enzyme acting on hydroperoxides and organic peroxides [216]. Distribution of genes encoding these enzymes is patchy within the Rhizobiaceae being present only in *R. rhizogenes* K84, *Rhizobium* sp. CIAT894, *R. leguminosarum* 3841 and WSM2304, and *A. vitis* S4.

A *R. tropici* CIAT 899 mutant deficient in glutathione biosynthesis is not only sensitive to acid stress, as previously mentioned, but also to oxidative stress [24]. We found one gene encoding a putative glutathione peroxidase (Gpo) in CIAT 899 and also in PRF 81. A low level of glutathione may affect the oxidative stress response as it compromise the activity of Gpo that uses glutathione as electron donor. In other bacteria, Gpo is involved in organic hydroperoxide resistance and virulence [217]. Glutathione also has a protective effect as it can directly reduce protein disulfide bonds induced by ROS. PRF 81 harbors a second glutathione synthase gene in its megaplasmid. Some agrobacteria and all sinorhizobia genomes also harbor a second glutathione synthase gene.

A gene encoding a member of the Dps family of ferritin proteins was identified in each genome. The ferroxidase activity of Dps proteins protects cells against oxidative damage by simultaneously removing from the cytosol H_2_O_2_ and free ferrous ion that otherwise react, producing hydroxil radicals by the Fenton reaction [218]. ROS induce protein damage by oxidation of methionine residues to methionine sulfoxides [219]. The CIAT 899 and PRF 81 genomes encoded for two MsrA and one MsrB methionine sulfoxide reductase repair-proteins [219].

Response to some oxidants like H_2_O_2_ may protect cells against osmotic and thermal stress [220]. Such overlap or cross-talk between the stress responses is not uncommon. We have recently found that the expression of several oxidative stress-related proteins of PRF 81 is up-regulated after exposure to high temperatures [184].

### Inorganic N assimilation and nitrosative stress

CIAT 899 and PRF 81 can use nitrate (NO_3_) as sole nitrogen source. Genome sequencing suggested that both strains can metabolize NO_3_ to ammonia via assimilatory nitrate reductase and nitrite reductase. Both strains possessed an ABC-type NO_3_-uptake transporter. PRF 81 seems able to use NO_3_ for respiration via a respiratory nitrate reductase and a NarK protein providing a bifunctional uptake NO_3_ symporter and NO_3_/nitrite antiporter. Neither CIAT 899 nor PRF 81 possessed genes for denitrification, i.e. NO_3_ reduction to N_2_. Denitrification is not a desirable characteristic of inoculant bacteria in agriculture as it causes loss of soil N.

Nitric oxide (NO) is a signaling and defense molecule in eukaryotes, and in the interaction between plant and microorganisms; host-plant cells respond to infection by generating NO [221]. NO is toxic to bacteria; it diffuses across cell membranes into the cytoplasm and reacts with iron centers and thiols causing so-called nitrosative stress [222]. PRF 81, but not CIAT 899, harbored a gene for the flavohemoglobin Hmp, a dioxygenase that catalyzes conversion of NO to NO_3_[223]. Bacterial flavohemoglobins are well-known scavengers of NO and play a crucial role in protecting animal pathogens from nitrosative stress during infection [224]. A *nsrR*-like transcriptional regulator gene, that in other bacteria is involved in nitrosative stress [225], was located adjacent to *hmp* and may regulate it. Both strain genomes possessed a homologue of the *E. coli* glutathione-dependent formaldehyde dehydrogenase, an enzyme protective against nitrosative stress due to its potent activity toward S-nitrosoglutathione, the condensation product of glutathione and NO [226]. NO may react with superoxide forming peroxynitrite, a potent oxidant, thus previously mentioned antioxidant proteins like AhpC/TSA and MsrA/MsrB also protect cells against nitrosative stress.

### Metal resistance

CIAT 899 and other *R. tropici* strains are more resistant to heavy metals than other bean-nodulating rhizobia [13]. This may be an advantageous trait for rhizobia growing in tropical acid soils where some of these metals can reach toxic concentrations. Genome analysis of CIAT 899 and PRF 81 revealed several genes that may be involved in metal resistance. Both strains possessed three genes coding for transporters of the cation diffusion facilitator (CDF) family. Different members of the CDF family are involved in efflux of Zn^+2^, Co^+2^, Cd^+2^, Ni^2+^, and less frequently, Mn^2+^, Fe^+2^, Cu^+2^ and mercury ions [227]. Additionally, 2 and 3 efflux P-type ATPases were identified in CIAT 899 and PRF 81, respectively. These transporters showed similarities to Zn-, Cd-, Au-, Ag- and Cu-efflux systems, including a protein required for resistance to Zn and Cd in *S. meliloti*[228]. Strain PRF 81 seems to be better equipped than CIAT 899 to contend with Cu excess, as it possessed three multicopper oxidase genes [229] and two Cu-sequestering protein *copD* genes [230] versus two and one of these systems, respectively, in CIAT 899. Interestingly, a homologue of the *cadD* gene required for Cd resistance in *S. aureus* was found in each genome [231]. *cadD* homologues are present mostly within the Firmicutes and the only other member of the Rhizobiales carrying a homologous gene was *R. rhizogenes* K84.

Both genomes carried a two-gene locus encoding a member of the chromate ion transporter (CHR) family and a regulator for chromate resistance [232]. Additionally, CIAT 899 genome had an alternative CHR efflux transporter system of the short-chain monodomain type [233]. Both strains had genes encoding the arsenic resistance proteins ArsC, which reduces arsenate to arsenite, and ArsB, which exports arsenite outside the cell [234]. CIAT899, but not PRF 81, had the tellurite resistance proteins TehA and TerC [235].

## Conclusions

Genome sequencing of the inoculant strains *R. tropici* CIAT 899 and *Rhizobium* sp. PRF 81 revealed that these rhizobia share a highly-conserved symbiotic plasmid. *R. leucaenae* CFN 299, showing a similar host range with CIAT 899 and PRF 81, also possessed this plasmid. The presence of this plasmid in three genetically different strains that are highly-efficient in N_2_-fixation with common bean highlights an evolution towards symbiotic effectiveness by the assembly of a successful set of nodulation and nitrogen-fixation genes in a pSym that has been spread and is maintained in geographically distant bacteria. In addition, this pSym carries genes for biosynthesis and modulation of plant-hormone levels, traits that are thought to improve the symbiotic or associative abilities of rhizobia.

The three divergent *nodA* genes present in the tropici symbiotic plasmid likely allow CIAT 899, PRF 81 and CFN 299 to have an expanded host range in comparison to rhizobia carrying a single gene. Recently, it has been described that *Rhizobium* strains isolated from nodules of *Mimosa* plants in French Guiana [236] and New Caledonia [237] each carries two different *nodA* genes, although they were distinct from the *nodA* genes present in the tropici pSym (Figure 4A). Symbiotic promiscuity seems to be a general characteristic of rhizobia [3] and our results suggest that reiteration of host-specific nodulation genes, like *nodA*, may be a previously unrecognized strategy to nodulate a wide range of legume hosts.

Competitiveness is a highly complex attribute that probably results from the combined activities of several classes of genes. CIAT 899 and PRF 81 possessed a wide array of genes coding for functions that may promote their competitiveness for root colonization and nodulation. Some traits such as the capacity to synthetize biotin and the NO-protecting enzyme Hmp coded in the PRF 81 genome but absent in CIAT 899 may explain the superior competitive ability of PRF 81.

The CIAT 899 and PRF 81 genomes evidence two rhizobial strains well equipped to cope with the environmental stress conditions found in tropical soils, including acidity and high temperatures, as well as oxidative and osmotic stresses. Proteins contributing to acid-stress response in other bacteria but not yet reported in rhizobia, such as a lipid A phosphoethanolamine transferase and a KcsA-like ion channel were found encoded in both genomes.

Remarkably, both genomes were found to be enriched in genes coding for drug-efflux pumps, a characteristic that helps to explain the resistance of CIAT 899 and PRF 81 to several antimicrobial compounds and probably also allows them to compete more effectively in the soil or rhizosphere against antimicrobial-producing microorganisms, or might even expand their legume host range by promoting resistance to antimicrobial compounds produced by their hosts.

## Methods

### Bacterial strains and growth conditions

*R. tropici* CIAT 899 was obtained from the culture collection of the Centro de Ciencias Genómicas, Cuernavaca, Mexico. *Rhizobium* sp. PRF 81 (=SEMIA 4088) and *R. leucaenae* CFN 299 were obtained from the Diazotrophic and Plant Growth Promoting Bacteria Collection of Embrapa Soja, Londrina, Paraná, Brazil. Main differences previously reported between the strains are shown in Additional file 2. Bacterial growth conditions and DNA extraction were performed as described before [32,238].

### Sequencing, assembly and gap closure

*R. tropici* CIAT 899 and *Rhizobium* sp. PRF 81 genomes were sequenced using a whole-genome shotgun strategy. For CIAT 899, a combination of Roche 454 GS-FLX shotgun and 3 kb-insert paired-end libraries were used at the sequencing facility of Virginia Bioinformatics Institute (USA). For PRF 81, a combination of GS-FLX shotgun libraries obtained at Creative Dynamics Inc. (USA) and Sanger sequences previously obtained at Embrapa Soja (Brazil) [32] were used. The pSym of *R. leucaenae* CFN 299 was transferred to a plasmid-less *Agrobacterium* strain and purified. The CFN 299 pSym DNA was Sanger sequenced at Embrapa Soja [32,238]. CFN 299 whole-genome sequence reads generated by Solid sequencing at Sistemas Genómicos (Spain) were used to complement sequencing of its pSym. For closing gaps, a primer walking strategy was used and PCR products were Sanger sequenced either at Embrapa Soja, or at Macrogen (Korea).

Roche 454 sequence reads were *de novo* assembled using Newbler. For PRF 81, Sanger reads were combined with pyrosequence data. Phrap [239] was used for *de novo* assembling of the Sanger reads of the CFN 299 pSym, and Solid sequences were added to the assembly using Bowtie [240]. Sanger reads obtained during the gap closure stage were added to the assemblies with the assistance of SeqManPro (DNASTAR) or Consed [241].

### Annotation

The genomes of CIAT 899 and PRF 81, and the pSym of CFN 299 were analyzed using the system for automated bacterial integrated annotation (SABIA) [242] and the rapid annotation using subsystem technology (RAST) server [243]. Both systems allowed the identification of gene-encoding regions, functional annotations, and manual curation of the gene annotations. Both systems also have tools for metabolic reconstruction using KEGG (Kyoto encyclopedia of genes and genomes) for sequence comparisons using BLAST and for functional comparisons using KEGG or FIGfam. The data for CIAT 899 and PRF 81 were submitted to the GenBank database and were assigned Bioprojects numbers PRJNA42391 and PRJNA13459, respectively. The data for the pSym of CFN 299 is available at the site http://www.bnf.lncc.br.

### Genome comparisons

Genome sequence and annotations from other bacteria were retrieved from the GenBank database. Genome alignments were performed with Mummer [244] and Mauve [245]. Whole genome relationships were depicted with a neighbor joining dendrogram constructed using a matrix of MUMi distances [38]. Orthologues were identified by the bidirectional best hit criterion using BLASTP with a minimum sequence identity of 70% measured over at least 70% of the length of the smallest protein. A systematic search for putative antimicrobial efflux pumps was initiated by scanning the proteome of each genome for sequences displaying PFAM domains characteristic of the MFS, RND, MATE and SMR (super)family transporters with HMMER3 [246]. To distinguish proteins involved in drug efflux from those transporting other kind of substrates, phylogenetic trees including all members of each super(family) deposited in the Transporter Classification Database [247] were constructed and only those clustering with known drug-efflux plumps were retained. Other genes belonging to functional classes described in this work were identified by searching for conserved motifs in the predicted proteomes and in the six-frame translations of the entire nucleotide sequence of each genome in order to avoid missing genes that were not predicted.

### Genetic manipulations and phenotypic characterizations

A 1541-bp fragment containing the complete *R. tropici* CIAT899 *waaL* gene was PCR amplified with Pfu Ultra polymerase (Stratagene) using primers 108F_orfL (5’-TGACCCAACGGCATAGGA-3’) and orfL_23R (5’-AGGCGGTCGTGCAACTA-3’). The PCR product was TA cloned into pCR4-TOPO, generating the plasmid pERN-T16b. To disrupt the gene by targeted insertional mutagenesis, an internal 467-bp *Eco*RV-*Apa*I fragment of *waaL* was obtained from pERN-T16b, blunted with T4 DNA polymerase and cloned into the suicide mobilizable vector pK18mob [248] digested with *Sma*I. The resulting plasmid, pERN-K1, was introduced into CIAT899 by conjugal transfer in a triparental mating using a helper strain containing the pRK2013 plasmid [249]. The CIAT899-E4 mutant was selected among the kanamycin-resistant transconjugants obtained after checking for proper pERN-K1 insertion by PCR using vector and *waaL* specific primers. For complementation experiments, the *waaL* cloned in pERN-T16b was subcloned as an *Eco*RI fragment into the expression vector pBBR1MCS-5 under the control of the *lacZ* promoter [250]. The orientation of the cloned gene was checked by PCR and restriction digestion. The selected plasmid, pERN-B10, was transferred into CIAT899-E4 as described above. LPS were isolated and visualized as previously described [30].

*R. leucaenae* CFN 299 and its 200-kb spontaneous pSym deletion mutant CFN299-10 [128] were grown in minimal medium supplied with tryptophan in the presence or absence of various flavonoid compounds. After 15 h of growth, cells were harvested and the supernatant treated for IAA extraction. The indole acidic fraction was extracted with ethyl acetate, dried under nitrogen flux and resuspended in methanol. Thin-layer chromatography was carried out and the IAA migration region was excised. IAA was extracted with methanol and quantified by HPLC.

## Competing interests

The authors declare that they have no competing interests.

## Authors’ contributions

EOO, EMR, MH and MM initiated and designed the study; EOO, PM, LMOC, JSSB and RCS performed the experiments; EOO, EMR, MH, PM, ASN, EPR, ATRV, LGPA, MFN, MM and FJO analyzed the data; MH, EMR, MM and ATRV contributed reagents/materials/analysis tools; EOO, EMR and MH wrote the paper. All authors read and approved the final manuscript.

## Supplementary Material

Additional file 1 **Diagram showing the number of orthologous gene clusters shared by *R. tropici *CIAT 899, *Rhizobium *sp. PRF 81, *R. rhizogenes *K84 and *R. etli *CFN 42.** Based on SABIA- and RAST-predicted genes. Click here for file

Additional file 2 Main characteristics of the three strains in this study. Click here for file

Additional file 3 **The WaaL O-antigen ligase of *Rhizobium tropici *CIAT 899.** A, Kite-Dolittle hydrophobicity plots of *Vibrio cholerae* V194 and *R. tropici* CIAT 899 WaaL proteins. B, *R. tropici* CIAT 899 WaaL protein represented as a light gray arrow with predicted membrane-spanning regions indicated with dark grey boxes and the position of the Pfam PF04932 conserved domain indicated with a white box. C, Multiple sequence alignment showing conserved regions shared by WaaL proteins. D, Lipolysaccharide profiles of *Rhizobium tropici* CIAT 899 (wt), its *waaL* mutant (E4), and the complemented mutant (E4 + *waaL*). Click here for file

Additional file 4 **Induction of indoleacetic acid (IAA) production by flavonoids in *R. leucaenae *CFN 299 and its 200-kb pSym deletion mutant CFN 299–10.** Click here for file

## References

[B1] Hungria M Stacey G Molecular signals exchanged between host plants and rhizobia: basic aspects and potential application in agriculture Soil Biol Biochem 1997 29 819 830 10.1016/S0038-0717(96)00239-8

[B2] Oldroyd GED Downie AJ Coordinating nodule morphogenesis with rhizobial infection in legumes Annu Rev Plant Biol 2008 59 519 546 10.1146/annurev.arplant.59.032607.092839 18444906

[B3] Perret X Staehelin C Broughton WJ Molecular basis of symbiotic promiscuity Microbiol Mol Biol Rev 2000 64 180 201 10.1128/MMBR.64.1.180-201.2000 10704479 PMC98991

[B4] Fauvart M Michiels J Rhizobial secreted proteins as determinants of host specificity in the rhizobium-legume symbiosis FEMS Microbiol Lett 2008 285 1 9 10.1111/j.1574-6968.2008.01254.x 18616593

[B5] Towe K Evolution of nitrogen fixation Science 2002 295 798 799 10.1126/science.295.5556.798 11824449

[B6] Raymond J Siefert JL Staples CR Blankenship RE The natural history of nitrogen fixation Mol Biol Evol 2004 21 541 554 14694078 10.1093/molbev/msh047

[B7] Martínez-Romero JC Ormeño-Orrillo E Rogel MA López-López A Martínez-Romero E Pontarotti P Trends in Rhizobial Evolution and Some Taxonomic Remarks Evolutionary Biology - Concepts, Molecular and Morphological Evolution 2010 Berlin Heidelberg: Springer 301 315

[B8] Gonzalez V Bustos P Ramirez-Romero MA Medrano-Soto A Salgado H Hernandez-Gonzalez I Hernandez-Celis JC Quintero V Moreno-Hagelsieb G Girard L The mosaic structure of the symbiotic plasmid of Rhizobium etli CFN42 and its relation to other symbiotic genome compartments Genome Biol 2003 4 R36 10.1186/gb-2003-4-6-r36 12801410 PMC193615

[B9] Ramsay JP Sullivan JT Jambari N Ortori CA Heeb S Williams P Barrett DA Lamont IL Ronson CW A LuxRI-family regulatory system controls excision and transfer of the Mesorhizobium loti strain R7A symbiosis island by activating expression of two conserved hypothetical genes Mol Microbiol 2009 73 1141 1155 10.1111/j.1365-2958.2009.06843.x 19682258

[B10] Galibert F Finan TM Long SR Puhler A Abola P Ampe F Barloy-Hubler F Barnett MJ Becker A Boistard P The composite genome of the legume symbiont Sinorhizobium meliloti Science 2001 293 668 672 10.1126/science.1060966 11474104

[B11] Kaneko T Nakamura Y Sato S Minamisawa K Uchiumi T Sasamoto S Watanabe A Idesawa K Iriguchi M Kawashima K Complete genomic sequence of nitrogen-fixing symbiotic bacterium Bradyrhizobium japonicum USDA110 DNA Res 2002 9 189 197 10.1093/dnares/9.6.189 12597275

[B12] Grange L Hungria M Graham PH Martínez-Romero E New insights into the origins and evolution of rhizobia that nodulate common bean (Phaseolus vulgaris) in Brazil Soil Biol Biochem 2007 39 867 876 10.1016/j.soilbio.2006.10.008

[B13] Martínez-Romero E Segovia L Mercante FM Franco AA Graham P Pardo MA Rhizobium tropici, a novel species nodulating Phaseolus vulgaris L. beans and Leucaena sp. trees Int J Syst Evol Microbiol 1991 41 417 426 10.1099/00207713-41-3-417 1715738

[B14] Ribeiro RA Rogel MA López-López A Ormeño-Orrillo E Gomes Barcellos F Martínez J Lopes Thompson F Martínez-Romero E Hungria M Reclassification of Rhizobium tropici type A strains as Rhizobium leucaenae sp. nov Int J Syst Evol Microbiol 2012 62 1180 1185 10.1099/ijs.0.032912-0 21742822

[B15] Hungria M Andrade DD Chueire LMD Probanza A Guttierrez-Manero FJ Megias M Isolation and characterization of new efficient and competitive bean (Phaseolus vulgaris L.) rhizobia from Brazil Soil Biol Biochem 2000 32 1515 1528 10.1016/S0038-0717(00)00063-8

[B16] Acosta-Durán C Martínez-Romero E Diversity of rhizobia from nodules of the leguminous tree Gliricidia sepium, a natural host of Rhizobium tropici Arch Microbiol 2002 178 161 164 10.1007/s00203-002-0433-3 12115061

[B17] Moron B Soria-Diaz ME Ault J Verroios G Noreen S Rodriguez-Navarro DN Gil-Serrano A Thomas-Oates J Megias M Sousa C Low pH changes the profile of nodulation factors produced by Rhizobium tropici CIAT899 Chem Biol 2005 12 1029 1040 10.1016/j.chembiol.2005.06.014 16183027

[B18] Rosenblueth M Martínez-Romero E Rhizobium etli maize populations and their competitiveness for root colonization Arch Microbiol 2004 181 337 344 10.1007/s00203-004-0661-9 15024554

[B19] Graham PH Draeger KJ Ferrey ML Conroy MJ Hammer BE Martinez E Aarons SR Quinto C Acid pH tolerance in strains of Rhizobium and Bradyrhizobium, and initial studies on the basis for acid tolerance of Rhizobium tropici UMR1899 Can J Microbiol/Rev Can Microbiol 1994 40 198 207 10.1139/m94-033

[B20] Pinto FGS Hungria M Mercante FM Polyphasic characterization of Brazilian Rhizobium tropici strains effective in fixing N2 with common bean (Phaseolus vulgaris L.) Soil Biol Biochem 2007 39 1851 1864 10.1016/j.soilbio.2007.01.001

[B21] Hungria M Campo RJ Mendes IC Benefits of inoculation of the common bean (Phaseolus vulgaris) crop with efficient and competitive Rhizobium tropici strains Biol Fertility Soils 2003 39 88 93 10.1007/s00374-003-0682-6

[B22] Hungria M Vargas MAT Campo RJ Chueire LMO Andrade DDS The Brazilian experience with the soybean (Glycine max) and common bean (Phaseolus vulgaris) symbioses Nitrogen Fixation: From Molecules to Crop Productivity 2000 38 515 518

[B23] Riccillo PM Collavino MM Grasso DH England R de Bruijn FJ Aguilar OM A guaB mutant strain of Rhizobium tropici CIAT899 pleiotropically defective in thermal tolerance and symbiosis Mol Plant Microbe Interact 2000 13 1228 1236 10.1094/MPMI.2000.13.11.1228 11059489

[B24] Riccillo PM Muglia CI de Bruijn FJ Roe AJ Booth IR Aguilar OM Glutathione is involved in environmental stress responses in Rhizobium tropici, including acid tolerance J Bacteriol 2000 182 1748 1753 10.1128/JB.182.6.1748-1753.2000 10692382 PMC94474

[B25] Rojas-Jimenez K Sohlenkamp C Geiger O Martínez-Romero E Werner D Vinuesa P A ClC chloride channel homolog and ornithine-containing membrane lipids of Rhizobium tropici CIAT899 are involved in symbiotic efficiency and acid tolerance Mol Plant Microbe Interact 2005 18 1175 1185 10.1094/MPMI-18-1175 16353552

[B26] Vences-Guzman MA Guan Z Ormeño-Orrillo E Gonzalez-Silva N Lopez-Lara IM Martínez-Romero E Geiger O Sohlenkamp C Hydroxylated ornithine lipids increase stress tolerance in Rhizobium tropici CIAT899 Mol Microbiol 2011 79 1496 1514 10.1111/j.1365-2958.2011.07535.x 21205018 PMC3053409

[B27] Vinuesa P Neumann-Silkow F Pacios-Bras C Spaink HP Martínez-Romero E Werner D Genetic analysis of a pH-regulated operon from Rhizobium tropici CIAT899 involved in acid tolerance and nodulation competitiveness Mol Plant Microbe Interact 2003 16 159 168 10.1094/MPMI.2003.16.2.159 12575750

[B28] Martínez-Romero E Segovia L Mercante FM Franco AA Graham P Pardo MA Rhizobium tropici, a novel species nodulating Phaseolus vulgaris L. beans and Leucaena sp. trees Int J Syst Bacteriol 1991 41 417 426 10.1099/00207713-41-3-417 1715738

[B29] Bernal GR Tlusty B de Jensen CE van Berkum P Graham PH Characteristics of rhizobia nodulating beans in the central region of Minnesota Can J Microbiol/Rev Can Microbiol 2004 50 1023 1031 10.1139/w04-092 15714233

[B30] Ormeño-Orrillo E Rosenblueth M Luyten E Vanderleyden J Martínez-Romero E Mutations in lipopolysaccharide biosynthetic genes impair maize rhizosphere and root colonization of Rhizobium tropici CIAT899 Environ Microbiol 2008 10 1271 1284 10.1111/j.1462-2920.2007.01541.x 18312393

[B31] Geniaux E Flores M Palacios R Martinez E Presence of megaplasmids in Rhizobium tropici and further evidence of differences between the two R. tropici subtypes Int J Syst Bacteriol 1995 45 392 394 10.1099/00207713-45-2-392

[B32] Pinto FGS Chueire LMO VasconcelosS ATR Nicolás MF Almeida LGP Souza RC Menna P Barcellos FG Megías M Hungria M Novel genes related to nodulation, secretion systems, and surface structures revealed by a genome draft of Rhizobium tropici strain PRF 81 Funct Integr Genomics 2009 9 263 270 10.1007/s10142-009-0109-z 19184146

[B33] Wood DW Setubal JC Kaul R Monks DE Kitajima JP Okura VK Zhou Y Chen L Wood GE Almeida NF Jr The genome of the natural genetic engineer Agrobacterium tumefaciens C58 Science 2001 294 2317 2323 10.1126/science.1066804 11743193

[B34] Ribeiro RA Barcellos FG Thompson FL Hungria M Multilocus sequence analysis of Brazilian Rhizobium microsymbionts of common bean (Phaseolus vulgaris L.) reveals unexpected taxonomic diversity Res Microbiol 2009 160 297 306 10.1016/j.resmic.2009.03.009 19403105

[B35] Slater SC Goldman BS Goodner B Setubal JC Farrand SK Nester EW Burr TJ Banta L Dickerman AW Paulsen I Genome sequences of three Agrobacterium biovars help elucidate the evolution of multichromosome genomes in bacteria J Bacteriol 2009 191 2501 2511 10.1128/JB.01779-08 19251847 PMC2668409

[B36] Velázquez E Palomo JL Rivas R Guerra H Peix A Trujillo ME García-Benavides P Mateos PF Wabiko H Martínez-Molina E Analysis of core genes supports the reclassification of strains Agrobacterium radiobacter K84 and Agrobacterium tumefaciens AKE10 into the species Rhizobium rhizogenes Syst Appl Microbiol 2010 33 247 251 10.1016/j.syapm.2010.04.004 20627641

[B37] Young JM Kuykendall LD Martínez-Romero E Kerr A Sawada H A revision of Rhizobium Frank 1889, with an emended description of the genus, and the inclusion of all species of Agrobacterium Conn 1942 and Allorhizobium undicola de Lajudie et al. 1998 as new combinations: Rhizobium radiobacter, R. rhizogenes, R. rubi, R. undicola and R. vitis Int J Syst Evol Microbiol 2001 51 89 103 11211278 10.1099/00207713-51-1-89

[B38] Deloger M El Karoui M Petit MA A genomic distance based on MUM indicates discontinuity between most bacterial species and genera J Bacteriol 2009 191 91 99 10.1128/JB.01202-08 18978054 PMC2612450

[B39] Auch AF von Jan M Klenk HP Goker M Digital DNA-DNA hybridization for microbial species delineation by means of genome-to-genome sequence comparison Stand Genomic Sci 2010 2 117 134 10.4056/sigs.531120 21304684 PMC3035253

[B40] Wayne LG Brenner DJ Colwell RR Grimont PAD Kandler O Krichevsky MI Moore LH Moore WEC Murray RGE Stackebrandt E Report of the ad hoc committee on reconciliation of approaches to bacterial systematics Int J Syst Bacteriol 1987 37 463 464 10.1099/00207713-37-4-463

[B41] Harrison PW Lower RP Kim NK Young JP Introducing the bacterial 'chromid': not a chromosome, not a plasmid Trends Microbiol 2010 18 141 148 10.1016/j.tim.2009.12.010 20080407

[B42] Crossman LC Castillo-Ramirez S McAnnula C Lozano L Vernikos GS Acosta JL Ghazoui ZF Hernandez-Gonzalez I Meakin G Walker AW A common genomic framework for a diverse assembly of plasmids in the symbiotic nitrogen fixing bacteria PLoS One 2008 3 e2567 10.1371/journal.pone.0002567 18596979 PMC2434198

[B43] Hungria M Andrade DS Chueire LMO Probanza A Guttierrez-Mañero FJ Megías M Isolation and characterization of new efficient and competitive bean (Phaseolus vulgaris L.) rhizobia from Brazil Soil Biol Biochem 2000 32 1515 1528 10.1016/S0038-0717(00)00063-8

[B44] Hernandez-Lucas I Ramirez-Trujillo JA Gaitan MA Guo X Flores M Martínez-Romero E Perez-Rueda E Mavingui P Isolation and characterization of functional insertion sequences of rhizobia FEMS Microbiol Lett 2006 261 25 31 10.1111/j.1574-6968.2006.00319.x 16842354

[B45] Lozano L Hernández-González I Bustos P Santamaría RI Souza V Young JPW Dávila G González V Evolutionary dynamics of insertion sequences in relation to the evolutionary histories of the chromosome and symbiotic plasmid genes of Rhizobium etli populations Appl Environ Microbiol 2010 76 6504 6513 10.1128/AEM.01001-10 20675442 PMC2950475

[B46] Rogel MA Ormeño-Orrillo E Martinez Romero E Symbiovars in rhizobia reflect bacterial adaptation to legumes Syst Appl Microbiol 2011 34 96 104 10.1016/j.syapm.2010.11.015 21306854

[B47] Valverde A Igual JM Peix A Cervantes E Velazquez E Rhizobium lusitanum sp. nov. a bacterium that nodulates Phaseolus vulgaris Int J Syst Evol Microbiol 2006 56 2631 2637 10.1099/ijs.0.64402-0 17082403

[B48] Poupot R Martínez-Romero E Prome JC Nodulation factors from Rhizobium tropici are sulfated or nonsulfated chitopentasaccharides containing an N-methyl-N-acylglucosaminyl terminus Biochemistry 1993 32 10430 10435 10.1021/bi00090a019 8399187

[B49] Folch-Mallol JL Marroqui S Sousa C Manyani H Lopez-Lara IM van der Drift KM Haverkamp J Quinto C Gil-Serrano A Thomas-Oates J Characterization of Rhizobium tropici CIAT899 nodulation factors: the role of nodH and nodPQ genes in their sulfation Mol Plant Microbe Interact 1996 9 151 163 10.1094/MPMI-9-0151 8850086

[B50] Waelkens F Voets T Vlassak K Vanderleyden J van Rhijn P The nodS gene of Rhizobium tropici strain CIAT899 is necessary for nodulation on Phaseolus vulgaris and on Leucaena leucocephala Mol Plant Microbe Interact 1995 8 147 154 10.1094/MPMI-8-0147 7772799

[B51] Vargas C Martinez LJ Megias M Quinto C Identification and cloning of nodulation genes and host specificity determinants of the broad host-range Rhizobium leguminosarum biovar phaseoli strain CIAT899 Mol Microbiol 1990 4 1899 1910 10.1111/j.1365-2958.1990.tb02039.x 2082147

[B52] Cárdenas L Dominguez J Santana O Quinto C The role of the nodI and nodJ genes in the transport of Nod metabolites in Rhizobium etli Gene 1996 173 183 187 10.1016/0378-1119(96)00166-7 8964496

[B53] Debelle F Plazanet C Roche P Pujol C Savagnac A Rosenberg C Prome JC Denarie J The NodA proteins of Rhizobium meliloti and Rhizobium tropici specify the N-acylation of Nod factors by different fatty acids Mol Microbiol 1996 22 303 314 10.1046/j.1365-2958.1996.00069.x 8930915

[B54] Rincon-Rosales R Lloret L Ponce E Martínez-Romero E Rhizobia with different symbiotic efficiencies nodulate Acaciella angustissima in Mexico, including Sinorhizobium chiapanecum sp. nov. which has common symbiotic genes with Sinorhizobium mexicanum FEMS Microbiol Ecol 2009 67 103 117 10.1111/j.1574-6941.2008.00590.x 19120461 PMC2784085

[B55] Yang G-P Debellé F Savagnac A Ferro M Schiltz O Maillet F Promé D Treilhou M Vialas C Lindstrom K Structure of theMesorhizobium huakuiiandRhizobium galegaeNod factors: a cluster of phylogenetically related legumes are nodulated by rhizobia producing Nod factors with α, β-unsaturated N-acyl substitutions. Mol Microbiol 1999 34 227 237 10.1046/j.1365-2958.1999.01582.x 10564467

[B56] Krishnan HB Chronis D Functional nodFE genes are present in Sinorhizobium sp. strain MUS10, a symbiont of the tropical legume Sesbania rostrata Appl Environ Microbiol 2008 74 2921 2923 10.1128/AEM.00075-08 18326678 PMC2394878

[B57] Hungria M Joseph CM Phillips DA Anthocyanidins and flavonols, major nod gene Inducers from seeds of a black-seeded common bean (Phaseolus vulgaris L.) Plant Physiol 1991 97 751 758 10.1104/pp.97.2.751 16668462 PMC1081070

[B58] Hungria M Joseph CM Phillips DA Rhizobium nod gene inducers exuded naturally from roots of common bean (Phaseolus vulgaris L.) Plant Physiol 1991 97 759 764 10.1104/pp.97.2.759 16668463 PMC1081071

[B59] van Rhijn PJ Feys B Verreth C Vanderleyden J Multiple copies of nodD in Rhizobium tropici CIAT899 and BR816 J Bacteriol 1993 175 438 447 8419293 10.1128/jb.175.2.438-447.1993 PMC196158

[B60] Sousa C Folch JL Boloix P Megias M Nava N Quinto C A Rhizobium tropici DNA region carrying the amino-terminal half of a nodD gene and a nod-box-like sequence confers host-range extension Mol Microbiol 1993 9 1157 1168 10.1111/j.1365-2958.1993.tb01245.x 7934929

[B61] van Rhijn PJ Desair J Vlassak K Vanderleyden J Functional analysis of nodD genes of Rhizobium tropici CIAT899 Mol Plant Microbe Interact 1994 7 666 677 10.1094/MPMI-7-0666

[B62] Estevez J Soria-Diaz ME de Cordoba FF Moron B Manyani H Gil A Thomas-Oates J van Brussel AA Dardanelli MS Sousa C Different and new Nod factors produced by Rhizobium tropici CIAT899 following Na+ stress FEMS Microbiol Lett 2009 293 220 231 10.1111/j.1574-6968.2009.01540.x 19260963

[B63] Baev N Schultze M Barlier I Ha DC Virelizier H Kondorosi E Kondorosi A RhizobiumnodMandnodNgenes are commonnodgenes:nodMencodes functions for efficiency of Nod signal production and bacteroid maturation. J Bacteriol 1992 174 7555 7565 1447128 10.1128/jb.174.23.7555-7565.1992 PMC207465

[B64] Bloemberg GV Thomas-Oates JE Lugtenberg BJJ Spaink HP Nodulation protein NodL of Rhizobium leguminosarum O-acetylates lipo-oligosaccharides, chitin fragments and N-acetylglucosamine in vitro Mol Microbiol 1994 11 793 804 10.1111/j.1365-2958.1994.tb00357.x 8196551

[B65] Kiss E Mergaert P Olàh B Kereszt A Staehelin C Davies AE Downie JA Kondorosi A Kondorosi E Conservation of noIR in the Sinorhizobium and Rhizobium genera of the Rhizobiaceae family Mol Plant Microbe Interact 1998 11 1186 1195 10.1094/MPMI.1998.11.12.1186

[B66] Masson-Boivin C Giraud E Perret X Batut J Establishing nitrogen-fixing symbiosis with legumes: how many rhizobium recipes? Trends Microbiol 2009 17 458 466 10.1016/j.tim.2009.07.004 19766492

[B67] Rubio LM Ludden PW Biosynthesis of the iron-molybdenum cofactor of nitrogenase Annu Rev Microbiol 2008 62 93 111 10.1146/annurev.micro.62.081307.162737 18429691

[B68] Edgren T Nordlund S Two pathways of electron transport to nitrogenase in Rhodospirillum rubrum: the major pathway is dependent on the fix gene products FEMS Microbiol Lett 2006 260 30 35 10.1111/j.1574-6968.2006.00297.x 16790015

[B69] Preisig O Zufferey R Hennecke H The Bradyrhizobium japonicum fixGHIS genes are required for the formation of the high-affinity cbb3-type cytochrome oxidase Arch Microbiol 1996 165 297 305 10.1007/s002030050330 8661920

[B70] Girard ML Flores M Brom S Romero D Palacios R Dávila G Structural complexity of the symbiotic plasmid of Rhizobium leguminosarum bv. phaseoli J Bacteriol 1991 173 2411 2419 2013564 10.1128/jb.173.8.2411-2419.1991 PMC207802

[B71] Soberón-Chávez G Nájera R Olivera H Segovia L Genetic rearrangements of a Rhizobium phaseoli symbiotic plasmid J Bacteriol 1986 167 487 491 3015875 10.1128/jb.167.2.487-491.1986 PMC212914

[B72] Mavingui P Laeremans T Flores M Romero D Martínez-Romero E Palacios R Genes essential for Nod factor production and nodulation are located on a symbiotic amplicon (AMPRtrCFN299pc60) in Rhizobium tropici J Bacteriol 1998 180 2866 2874 9603874 10.1128/jb.180.11.2866-2874.1998 PMC107251

[B73] Hungria M Neves MCP Cultivar and Rhizobium strain effect on nitrogen fixation and transport in Phaseolus vulgaris L Plant Soil 1987 103 111 121 10.1007/BF02370675

[B74] Schubert KR Evans HJ Hydrogen evolution: A major factor affecting the efficiency of nitrogen fixation in nodulated symbionts Proc Natl Acad Sci USA 1976 73 1207 1211 10.1073/pnas.73.4.1207 16592307 PMC430231

[B75] Baginsky C Brito B Imperial J Palacios J-M Ruiz-Argüeso T Diversity and evolution of dydrogenase systems in rhizobia Appl Environ Microbiol 2002 68 4915 4924 10.1128/AEM.68.10.4915-4924.2002 12324339 PMC126442

[B76] van Berkum P Navarro RB Vargas AAT Classification of the uptake hydrogenase-positive (Hup+) bean rhizobia as Rhizobium tropici Appl Environ Microbiol 1994 60 554 561 8135515 10.1128/aem.60.2.554-561.1994 PMC201348

[B77] Manyani H Rey L Palacios JM Imperial J Ruiz-Argüeso T Gene products of the hupGHIJ operon are involved in maturation of the iron-sulfur subunit of the [NiFe] hydrogenase from Rhizobium leguminosarum bv. viciae J Bacteriol 2005 187 7018 7026 10.1128/JB.187.20.7018-7026.2005 16199572 PMC1251625

[B78] Brito B Prieto R-I Cabrera E Mandrand-Berthelot M-A Imperial J Ruiz-Argüeso T Palacios J-M Rhizobium leguminosarum hupE encodes a nickel transporter required for hydrogenase activity J Bacteriol 2010 192 925 935 10.1128/JB.01045-09 20023036 PMC2812973

[B79] Becker A Fraysse N Sharypova L Recent advances in studies on structure and symbiosis-related function of rhizobial K-antigenss and lipopolysaccharides. Mol Plant Microbe Interact 2005 18 899 905 10.1094/MPMI-18-0899 16167760

[B80] Skorupska A Janczarek M Marczak M Mazur A Krol J Rhizobial exopolysaccharides: genetic control and symbiotic functions Microb Cell Fact 2006 5 7 10.1186/1475-2859-5-7 16483356 PMC1403797

[B81] D'Haeze W Holsters M Surface polysaccharides enable bacteria to evade plant immunity Trends Microbiol 2004 12 555 561 10.1016/j.tim.2004.10.009 15539115

[B82] Ingram BO Sohlenkamp C Geiger O Raetz CRH Altered lipid A structures and polymyxin hypersensitivity of Rhizobium etli mutants lacking the LpxE and LpxF phosphatases Biochim Biophys Acta 2010 1801 593 604 10.1016/j.bbalip.2010.02.001 20153447 PMC2839054

[B83] Reuhs BL Kim JS Matthysse AG Attachment of Agrobacterium tumefaciens to carrot cells and Arabidopsis wound sites is correlated with the presence of a cell-associated, acidic polysaccharide J Bacteriol 1997 179 5372 5379 9286990 10.1128/jb.179.17.5372-5379.1997 PMC179406

[B84] Kiss E Reuhs BL Kim JS Kereszt A Petrovics G Putnoky P Dusha I Carlson RW Kondorosi A The rkpGHI and -J genes are involved in capsular polysaccharide production by Rhizobium meliloti J Bacteriol 1997 179 2132 2140 9079896 10.1128/jb.179.7.2132-2140.1997 PMC178947

[B85] Petrovics G Putnoky P Reuhs B Kim J Thorp TA Noel KD Carlson RW Kondorosi A The presence of a novel type of surface polysaccharide in Rhizobium meliloti requires a new fatty acid synthase-like gene cluster involved in symbiotic nodule development Mol Microbiol 1993 8 1083 1094 10.1111/j.1365-2958.1993.tb01653.x 8361353

[B86] Hidalgo A Margaret I Crespo-Rivas JC Parada M Murdoch PS López A Buendía-Clavería AM Moreno J Albareda M Gil-Serrano AM The rkpU gene of Sinorhizobium fredii HH103 is required for bacterial K-antigen polysaccharide production and for efficient nodulation with soybean but not with cowpea Microbiology 2010 156 3398 3411 10.1099/mic.0.042499-0 20688828

[B87] Kereszt A Kiss E Reuhs BL Carlson RW Kondorosi A Putnoky P Novel rkp gene clusters of Sinorhizobium meliloti involved in capsular polysaccharide production and invasion of the symbiotic nodule: the rkpK gene encodes a UDP-glucose dehydrogenase J Bacteriol 1998 180 5426 5431 9765575 10.1128/jb.180.20.5426-5431.1998 PMC107592

[B88] Laus MC Logman TJ van Brussel AAN Carlson RW Azadi P Gao M-Y Kijne JW Involvement of exo5 in production of surface polysaccharides in Rhizobium leguminosarum and its role in nodulation of Vicia sativa subsp. nigra J Bacteriol 2004 186 6617 6625 10.1128/JB.186.19.6617-6625.2004 15375143 PMC516619

[B89] Abarca-Grau AM Burbank LP de Paz HD Crespo-Rivas JC Marco-Noales E López MM Vinardell JM von Bodman SB Penyalver R Role for Rhizobium rhizogenes K84 cell envelope polysaccharides in surface interactions Appl Environ Microbiol 2012 78 1644 1651 10.1128/AEM.07117-11 22210213 PMC3298171

[B90] Gil-Serrano A del Junco AS Tejero-Mateo P Megias M Caviedes MA Structure of the extracellular polysaccharide secreted by Rhizobium leguminosarum var. phaseoli CIAT 899 Carbohydr Res 1990 204 103 107 2279241 10.1016/0008-6215(90)84025-p

[B91] Leigh JA Reed JW Hanks JF Hirsch AM Walker GC Rhizobium meliloti mutants that fail to succinylate their calcofluor-binding exopolysaccharide are defective in nodule invasion Cell 1987 51 579 587 10.1016/0092-8674(87)90127-9 2824062

[B92] Milner JL Araujo RS Handelsman J Molecular and symbiotic characterization of exopolysaccharide-deficient mutants of Rhizobium tropici strain CIAT899 Mol Microbiol 1992 6 3137 3147 10.1111/j.1365-2958.1992.tb01770.x 1453954

[B93] Glucksmann MA Reuber TL Walker GC Genes needed for the modification, polymerization, export, and processing of succinoglycan byRhizobium meliloti: a model for succinoglycan biosynthesis J Bacteriol 1993 175 7045 7055 8226646 10.1128/jb.175.21.7045-7055.1993 PMC206832

[B94] Ormeño-Orrillo E Lipopolysaccharides of rhizobiaceae: structure and biosynthesis Rev Latinoam Microbiol 2005 47 165 175 17061540

[B95] Raetz CR Reynolds CM Trent MS Bishop RE Lipid A modification systems in Gram-negative bacteria Annu Rev Biochem 2007 76 295 329 10.1146/annurev.biochem.76.010307.145803 17362200 PMC2569861

[B96] Sharypova LA Niehaus K Scheidle H Holst O Becker A Sinorhizobium meliloti acpXL mutant lacks the C28 hydroxylated fatty acid moiety of lipid A and does not express a slow migrating form of lipopolysaccharide J Biol Chem 2003 278 12946 12954 10.1074/jbc.M209389200 12566460

[B97] Vedam V Kannenberg EL Haynes JG Sherrier DJ Datta A Carlson RW A Rhizobium leguminosarum AcpXL mutant produces lipopolysaccharide lacking 27-hydroxyoctacosanoic acid J Bacteriol 2003 185 1841 1850 10.1128/JB.185.6.1841-1850.2003 12618448 PMC150140

[B98] Gil-Serrano AM Gonzalez-Jimenez I Tejero-Mateo P Megias M Romero-Vazquez MJ Analysis of the lipid moiety of lipopolysaccharide from Rhizobium tropici CIAT899: identification of 29-hydroxytriacontanoic acid J Bacteriol 1994 176 2454 2457 8157617 10.1128/jb.176.8.2454-2457.1994 PMC205373

[B99] Kadrmas JL Allaway D Studholme RE Sullivan JT Ronson CW Poole PS Raetz CR Cloning and overexpression of glycosyltransferases that generate the lipopolysaccharide core of Rhizobium leguminosarum J Biol Chem 1998 273 26432 26440 10.1074/jbc.273.41.26432 9756877

[B100] Gil-Serrano AM Gonzalez-Jimenez I Tejero Mateo P Bernabe M Jimenez-Barbero J Megias M Romero-Vazquez MJ Structural analysis of the O-antigen of the lipopolysaccharide of Rhizobium tropici CIAT899 Carbohydr Res 1995 275 285 294 10.1016/0008-6215(95)00178-V 8529224

[B101] Gerlach RG Hensel M Protein secretion systems and adhesins: the molecular armory of Gram-negative pathogens Int J Med Microbiol 2007 297 401 415 10.1016/j.ijmm.2007.03.017 17482513

[B102] Deakin WJ Broughton WJ Symbiotic use of pathogenic strategies: rhizobial protein secretion systems Nat Rev Micro 2009 7 312 320 10.1038/nrmicro2091 19270720

[B103] Thanabalu T Koronakis E Hughes C Koronakis V Substrate-induced assembly of a contiguous channel for protein export from E.coli: reversible bridging of an inner-membrane translocase to an outer membrane exit pore EMBO J 1998 17 6487 6496 10.1093/emboj/17.22.6487 9822594 PMC1170996

[B104] Cosme AM Becker A Santos MR Sharypova LA Santos PM Moreira LM The outer membrane protein TolC from Sinorhizobium meliloti affects protein secretion, polysaccharide biosynthesis, antimicrobial resistance, and symbiosis Mol Plant Microbe Interact 2008 21 947 957 10.1094/MPMI-21-7-0947 18533835

[B105] Linhartova I Bumba L Masin J Basler M Osicka R Kamanova J Prochazkova K Adkins I Hejnova-Holubova J Sadilkova L RTX proteins: a highly diverse family secreted by a common mechanism FEMS Microbiol Rev 2010 34 1076 1112 20528947 10.1111/j.1574-6976.2010.00231.x PMC3034196

[B106] York GM Walker GC The Rhizobium meliloti exoK gene and prsD/prsE/exsH genes are components of independent degradative pathways which contribute to production of low-molecular-weight succinoglycan Mol Microbiol 1997 25 117 134 10.1046/j.1365-2958.1997.4481804.x 11902715

[B107] Oresnik IJ Twelker S Hynes MF Cloning and characterization of a Rhizobium leguminosarum gene encoding a bacteriocin with similarities to RTX toxins Appl Environ Microbiol 1999 65 2833 2840 10388672 10.1128/aem.65.7.2833-2840.1999 PMC91425

[B108] Hayes CS Aoki SK Low DA Bacterial contact-dependent delivery systems Annu Rev Genet 2010 44 71 90 10.1146/annurev.genet.42.110807.091449 21047256

[B109] Relman DA Domenighini M Tuomanen E Rappuoli R Falkow S Filamentous hemagglutinin of Bordetella pertussis: nucleotide sequence and crucial role in adherence Proc Natl Acad Sci USA 1989 86 2637 2641 10.1073/pnas.86.8.2637 2539596 PMC286972

[B110] Gottig N Garavaglia BS Garofalo CG Orellano EG Ottado J A filamentous hemagglutinin-like protein of Xanthomonas axonopodis pv. citri, the phytopathogen responsible for citrus canker, is involved in bacterial virulence PLoS One 2009 4 e4358 10.1371/journal.pone.0004358 19194503 PMC2632755

[B111] El Tahir Y Skurnik M YadA, the multifaceted Yersinia adhesin Int J Med Microbiol 2001 291 209 218 10.1078/1438-4221-00119 11554561

[B112] Juhas M Crook DW Hood DW Type IV secretion systems: tools of bacterial horizontal gene transfer and virulence Cell Microbiol 2008 10 2377 2386 10.1111/j.1462-5822.2008.01187.x 18549454 PMC2688673

[B113] McClure NC Ahmadi A-R Clare BG Construction of a range of derivatives of the biological control strain Agrobacterium rhizogenes K84: a study of factors involved in biological control of crown gall disease Appl Environ Microbiol 1998 64 3977 3982 9758829 10.1128/aem.64.10.3977-3982.1998 PMC106588

[B114] Christie PJ Atmakuri K Krishnamoorthy V Jakubowski S Cascales E Biogenesis, architecture, and function of bacterial type IV secretion systems Annu Rev Microbiol 2005 59 451 485 10.1146/annurev.micro.58.030603.123630 16153176 PMC3872966

[B115] Hubber A Vergunst AC Sullivan JT Hooykaas PJ Ronson CW Symbiotic phenotypes and translocated effector proteins of the Mesorhizobium loti strain R7A VirB/D4 type IV secretion system Mol Microbiol 2004 54 561 574 10.1111/j.1365-2958.2004.04292.x 15469524

[B116] Hubber AM Sullivan JT Ronson CW Symbiosis-induced cascade regulation of the Mesorhizobium loti R7A VirB/D4 type IV secretion system Mol Plant Microbe Interact 2007 20 255 261 10.1094/MPMI-20-3-0255 17378428

[B117] Jones KM Lloret J Daniele JR Walker GC The Type IV secretion system of Sinorhizobium meliloti strain 1021 is required for conjugation but not for intracellular symbiosis J Bacteriol 2007 189 2133 2138 10.1128/JB.00116-06 17158676 PMC1855733

[B118] Pérez-Mendoza D Sepúlveda E Pando V Muñoz S Nogales J Olivares J Soto MJ Herrera-Cervera JA Romero D Brom S Identification of the rctA gene, which is required for repression of conjugative transfer of rhizobial symbiotic megaplasmids J Bacteriol 2005 187 7341 7350 10.1128/JB.187.21.7341-7350.2005 16237017 PMC1272987

[B119] Tun-Garrido C Bustos P González V Brom S Conjugative transfer of p42a from Rhizobium etli CFN42, which Is required for mobilization of the symbiotic plasmid, is regulated by quorum sensing J Bacteriol 2003 185 1681 1692 10.1128/JB.185.5.1681-1692.2003 12591886 PMC148057

[B120] Wibberg D Blom J Jaenicke S Kollin F Rupp O Scharf B Schneiker-Bekel S Sczcepanowski R Goesmann A Setubal JC Complete genome sequencing ofAgrobacteriumsp. H13-3, the formerRhizobiumlupiniH13-3, reveals a tripartite genome consisting of a circular and a linear chromosome and an accessory plasmid but lacking a tumor-inducing Ti-plasmid. J Biotechnol 2011 155 50 62 10.1016/j.jbiotec.2011.01.010 21329740

[B121] Johnston AWB Todd JD Curson AR Lei S Nikolaidou-Katsaridou N Gelfand MS Rodionov DA Living without Fur: the subtlety and complexity of iron-responsive gene regulation in the symbiotic bacterium Rhizobium and other alpha-proteobacteria Biometals 2007 20 501 511 10.1007/s10534-007-9085-8 17310401

[B122] Carter RA Worsley PS Sawers G Challis GL Dilworth MJ Carson KC Lawrence JA Wexler M Johnston AWB Yeoman KH The vbs genes that direct synthesis of the siderophore vicibactin in Rhizobium leguminosarum: their expression in other genera requires ECF σ factor RpoI Mol Microbiol 2002 44 1153 1166 10.1046/j.1365-2958.2002.02951.x 12028377

[B123] Krause A Ramakumar A Bartels D Battistoni F Bekel T Boch J Bohm M Friedrich F Hurek T Krause L Complete genome of the mutualistic, N2-fixing grass endophyte Azoarcus sp. strain BH72 Nat Biotechnol 2006 24 1385 1391 10.1038/nbt1243 17057704

[B124] López C Crosa J Characterization of ferric-anguibactin transport in Vibrio anguillarum Biometals 2007 20 393 403 10.1007/s10534-007-9084-9 17287889

[B125] Amarelle V O'Brian MR Fabiano E ShmR is essential for utilization of heme as a nutritional iron source in Sinorhizobium meliloti Appl Environ Microbiol 2008 74 6473 6475 10.1128/AEM.01590-08 18757569 PMC2570276

[B126] Wexler M Yeoman KH Stevens JB De Luca NG Sawers G Johnston AWB The Rhizobium leguminosarum tonB gene is required for the uptake of siderophore and haem as sources of iron Mol Microbiol 2001 41 801 816 11532145 10.1046/j.1365-2958.2001.02556.x

[B127] Guerinot ML Meidl EJ Plessner O Citrate as a siderophore in Bradyrhizobium japonicum J Bacteriol 1990 172 3298 3303 2140566 10.1128/jb.172.6.3298-3303.1990 PMC209139

[B128] Pardo MA Lagunez J Miranda J Martinez E Nodulating ability of Rhizobium tropici is conditioned by a plasmid-encoded citrate synthase Mol Microbiol 1994 11 315 321 10.1111/j.1365-2958.1994.tb00311.x 8170393

[B129] Ding Y Oldroyd GED Positioning the nodule, the hormone dictum Plant Signal Behav 2009 4 89 93 10.4161/psb.4.2.7693 19649179 PMC2637488

[B130] Glick BR Modulation of plant ethylene levels by the bacterial enzyme ACC deaminase FEMS Microbiol Lett 2005 251 1 7 10.1016/j.femsle.2005.07.030 16099604

[B131] Conforte VP Echeverria M Sánchez C Ugalde RA Menéndez AV Lepek VC Engineered ACC deaminase-expressing free-living cells of Mesorhizobium loti show increased nodulation efficiency and competitiveness on Lotus spp J Gen Appl Microbiol 2010 56 331 338 10.2323/jgam.56.331 20953097

[B132] Tittabutr P Awaya JD Li QX Borthakur D The cloned 1-aminocyclopropane-1-carboxylate (ACC) deaminase gene from Sinorhizobium sp. strain BL3 in Rhizobium sp. strain TAL1145 promotes nodulation and growth of Leucaena leucocephala Syst Appl Microbiol 2008 31 141 150 10.1016/j.syapm.2008.03.001 18406559

[B133] Ma W Guinel FC Glick BR Rhizobium leguminosarum biovar viciae 1-aminocyclopropane-1-carboxylate deaminase promotes nodulation of pea plants Appl Environ Microbiol 2003 69 4396 4402 10.1128/AEM.69.8.4396-4402.2003 12902221 PMC169147

[B134] Spaepen S Vanderleyden J Remans R Indole-3-acetic acid in microbial and microorganism-plant signaling FEMS Microbiol Rev 2007 31 425 448 10.1111/j.1574-6976.2007.00072.x 17509086

[B135] Bonnard G Vincent F Otten L Sequence of Agrobacterium tumefaciens biotype III auxin genes Plant Mol Biol 1991 16 733 738 10.1007/BF00023438 1868204

[B136] Theunis M Kobayashi H Broughton WJ Prinsen E Flavonoids, NodD1, NodD2, and nod-box NB15 modulate expression of the y4wEFG locus that is required for indole-3-acetic acid synthesis in Rhizobium sp. strain NGR234 Mol Plant Microbe Interact 2004 17 1153 1161 10.1094/MPMI.2004.17.10.1153 15497408

[B137] Morrone D Chambers J Lowry L Kim G Anterola A Bender K Peters RJ Gibberellin biosynthesis in bacteria: separate ent-copalyl diphosphate and ent-kaurene synthases in Bradyrhizobium japonicum FEBS Lett 2009 583 475 480 10.1016/j.febslet.2008.12.052 19121310

[B138] Li XZ Nikaido H Efflux-mediated drug resistance in bacteria: an update Drugs 2009 69 1555 1623 10.2165/11317030-000000000-00000 19678712 PMC2847397

[B139] Holden MTG Seth-Smith HMB Crossman LC Sebaihia M Bentley SD Cerdeño-Tárraga AM Thomson NR Bason N Quail MA Sharp S The genome of Burkholderia cenocepacia J2315, an epidemic pathogen of cystic fibrosis patients J Bacteriol 2009 191 261 277 10.1128/JB.01230-08 18931103 PMC2612433

[B140] D’Costa VM Griffiths E Wright GD Expanding the soil antibiotic resistome: exploring environmental diversity Curr Opin Microbiol 2007 10 481 489 10.1016/j.mib.2007.08.009 17951101

[B141] Hibbing ME Fuqua C Parsek MR Peterson SB Bacterial competition: surviving and thriving in the microbial jungle Nat Rev Micro 2010 8 15 25 10.1038/nrmicro2259 PMC2879262 19946288

[B142] Butcher BG Helmann JD Identification of Bacillus subtilis σW-dependent genes that provide intrinsic resistance to antimicrobial compounds produced by bacilli Mol Microbiol 2006 60 765 782 10.1111/j.1365-2958.2006.05131.x 16629676

[B143] González-Lamothe R Mitchell G Gattuso M Diarra M Malouin F Bouarab K Plant antimicrobial agents and their effects on plant and human pathogens Int J Mol Sci 2009 10 3400 3419 10.3390/ijms10083400 20111686 PMC2812829

[B144] Haag AF Baloban M Sani M Kerscher B Pierre O Farkas A Longhi R Boncompagni E Hérouart D Dall’Angelo S Protection of Sinorhizobium against host cysteine-rich antimicrobial peptides is critical for symbiosis PLoS Biol 2011 9 e1001169 10.1371/journal.pbio.1001169 21990963 PMC3186793

[B145] Bhattacharya A Sood P Citovsky V The roles of plant phenolics in defence and communication during Agrobacterium and Rhizobium infection Mol Plant Pathol 2010 11 705 719 20696007 10.1111/j.1364-3703.2010.00625.x PMC6640454

[B146] Gonzalez-Pasayo R Martínez-Romero E Multiresistance genes of Rhizobium etli CFN42 Mol Plant Microbe Interact 2000 13 572 577 10.1094/MPMI.2000.13.5.572 10796024

[B147] Lindemann A Koch M Pessi G Muller AJ Balsiger S Hennecke H Fischer HM Host-specific symbiotic requirement of BdeAB, a RegR-controlled RND-type efflux system in Bradyrhizobium japonicum FEMS Microbiol Lett 2010 312 184 191 10.1111/j.1574-6968.2010.02115.x 20883496

[B148] Proft T Baker EN Pili in Gram-negative and Gram-positive bacteria - structure, assembly and their role in disease Cell Mol Life Sci 2009 66 613 635 10.1007/s00018-008-8477-4 18953686 PMC11131518

[B149] Stemmer WPC Sequeira L Fimbriae of phytopathogenic and symbiotic bacteria Phytopathology 1987 77 1633 1639 10.1094/Phyto-77-1633

[B150] Vesper SJ Bauer WD Role of pili (fimbriae) in attachment of Bradyrhizobium japonicum to soybean roots Appl Environ Microbiol 1986 52 134 141 16347100 10.1128/aem.52.1.134-141.1986 PMC203409

[B151] Dorr J Hurek T Reinhold-Hurek B Type IV pili are involved in plant-microbe and fungus-microbe interactions Mol Microbiol 1998 30 7 17 10.1046/j.1365-2958.1998.01010.x 9786181

[B152] Miller LD Yost CK Hynes MF Alexandre G The major chemotaxis gene cluster of Rhizobium leguminosarum bv. viciae is essential for competitive nodulation Mol Microbiol 2007 63 348 362 17163982 10.1111/j.1365-2958.2006.05515.x

[B153] Lippincott JA Beiderbeck R Lippincott BB Utilization of octopine and nopaline by Agrobacterium J Bacteriol 1973 116 378 383 4745420 10.1128/jb.116.1.378-383.1973 PMC246433

[B154] Oger P Mansouri H Dessaux Y Effect of crop rotation and soil cover on alteration of the soil microflora generated by the culture of transgenic plants producing opines Mol Ecol 2000 9 881 890 10.1046/j.1365-294x.2000.00940.x 10886651

[B155] Richardson JS Hynes MF Oresnik IJ A genetic locus necessary for rhamnose uptake and catabolism in Rhizobium leguminosarum bv. trifolii J Bacteriol 2004 24 8433 8442 15576793 10.1128/JB.186.24.8433-8442.2004 PMC532407

[B156] Fry J Wood M Poole PS Investigation of myo-inositol catabolism in Rhizobium leguminosarum bv. viciae and its effect on nodulation competitiveness Mol Plant Microbe Interact 2001 14 1016 1025 10.1094/MPMI.2001.14.8.1016 11497462

[B157] Rosenblueth M Hynes MF Martínez-Romero E Rhizobium tropici teu genes involved in specific uptake of Phaseolus vulgaris bean-exudate compounds Mol Gen Genet 1998 258 587 598 10.1007/s004380050772 9671027

[B158] Bittinger MA Handelsman J Identification of genes in the RosR regulon of Rhizobium etli J Bacteriol 2000 182 1706 1713 10.1128/JB.182.6.1706-1713.2000 10692377 PMC94469

[B159] Coleman SA Minnick MF Establishing a direct role for the Bartonella bacilliformis invasion-associated locus B (ialB) protein in human erythrocyte parasitism Infect Immun 2001 69 4373 4381 10.1128/IAI.69.7.4373-4381.2001 11401976 PMC98509

[B160] Smit G Kijne JW Lugtenberg BJ Involvement of both cellulose fibrils and a Ca2+-dependent adhesin in the attachment of Rhizobium leguminosarum to pea root hair tips J Bacteriol 1987 169 4294 4301 3624205 10.1128/jb.169.9.4294-4301.1987 PMC213743

[B161] Robledo M Jiménez-Zurdo JI Velázquez E Trujillo ME Zurdo-Piñeiro JL Ramírez-Bahena MH Ramos B Díaz-Mínguez JM Dazzo F Martínez-Molina E Rhizobium cellulase CelC2 is essential for primary symbiotic infection of legume host roots Proc Natl Acad Sci USA 2008 105 7064 7069 10.1073/pnas.0802547105 18458328 PMC2383954

[B162] Reinhold-Hurek B Maes T Gemmer S Van Montagu M Hurek T An endoglucanase is involved in infection of rice roots by the not-cellulose-metabolizing endophyte Azoarcus sp. strain BH72 Mol Plant Microbe Interact 2006 19 181 188 10.1094/MPMI-19-0181 16529380

[B163] Guillén-Navarro K Encarnación S Dunn MF Biotin biosynthesis, transport and utilization in rhizobia FEMS Microbiol Lett 2005 246 159 165 10.1016/j.femsle.2005.04.020 15899401

[B164] Sohlenkamp C Galindo-Lagunas KA Guan Z Vinuesa P Robinson S Thomas-Oates J Raetz CR Geiger O The lipid lysyl-phosphatidylglycerol is present in membranes of Rhizobium tropici CIAT899 and confers increased resistance to polymyxin B under acidic growth conditions Mol Plant Microbe Interact 2007 20 1421 1430 10.1094/MPMI-20-11-1421 17977153

[B165] Correa OS Rivas EA Barneix AJ Cellular envelopes and tolerance to acid pH in Mesorhizobium loti Curr Microbiol 1999 38 329 334 10.1007/PL00006812 10341073

[B166] Jordan KN Oxford L O’Byrne CP Survival of low-pH stress by Escherichia coli O157:H7: correlation between alterations in the cell envelope and increased acid tolerance Appl Environ Microbiol 1999 65 3048 3055 10388702 10.1128/aem.65.7.3048-3055.1999 PMC91455

[B167] Ballen KG Graham PH Jones RK Bowers JH Acidity and calcium interaction affecting cell envelope stability in Rhizobium Can J Microbiol/Rev Can Microbiol 1998 44 582 587

[B168] Martinic M Hoare A Contreras I Alvarez SA Contribution of the lipopolysaccharide to resistance of Shigella flexneri 2a to extreme acidity PLoS One 2011 6 e25557 10.1371/journal.pone.0025557 21984920 PMC3184986

[B169] Shabala L Ross T Cyclopropane fatty acids improve Escherichia coli survival in acidified minimal media by reducing membrane permeability to H+ and enhanced ability to extrude H+ Res Microbiol 2008 159 458 461 10.1016/j.resmic.2008.04.011 18562182

[B170] Yuan ZC Liu P Saenkham P Kerr K Nester EW Transcriptome profiling and functional analysis of Agrobacterium tumefaciens reveals a general conserved response to acidic conditions (pH 5.5) and a complex acid-mediated signaling involved in Agrobacterium-plant interactions J Bacteriol 2008 190 494 507 10.1128/JB.01387-07 17993523 PMC2223696

[B171] Hellweg C Puhler A Weidner S The time course of the transcriptomic response of Sinorhizobium meliloti 1021 following a shift to acidic pH BMC Microbiol 2009 9 37 10.1186/1471-2180-9-37 19216801 PMC2651895

[B172] Cunningham SD Munns DN The correlation between extracellular polysaccharide production and acid tolerance in Rhizobium Soil Sci Soc Am J 1984 48 1273 1276 10.2136/sssaj1984.03615995004800060014x

[B173] Foster JW Escherichia coli acid resistance: tales of an amateur acidophile Nat Rev Microbiol 2004 2 898 907 10.1038/nrmicro1021 15494746

[B174] Muglia CI Grasso DH Aguilar OM Rhizobium tropici response to acidity involves activation of glutathione synthesis Microbiology 2007 153 1286 1296 10.1099/mic.0.2006/003483-0 17379738

[B175] Accardi A Miller C Secondary active transport mediated by a prokaryotic homologue of ClC Cl- channels Nature 2004 427 803 807 10.1038/nature02314 14985752

[B176] Putnoky P Kereszt A Nakamura T Endre G Grosskopf E Kiss P Kondorosi Á The pha gene cluster of Rhizobium meliloti involved in pH adaptation and symbiosis encodes a novel type of K+ efflux system Mol Microbiol 1998 28 1091 1101 10.1046/j.1365-2958.1998.00868.x 9680201

[B177] Yang L Jiang J Wei W Zhang B Wang L Yang S The pha2 gene cluster involved in Na + resistance and adaption to alkaline pH in Sinorhizobium fredii RT19 encodes a monovalent cation/proton antiporter FEMS Microbiol Lett 2006 262 172 177 10.1111/j.1574-6968.2006.00385.x 16923072

[B178] Minder AC Fischer HM Hennecke H Narberhaus F Role of HrcA and CIRCE in the heat shock regulatory network of Bradyrhizobium japonicum J Bacteriol 2000 182 14 22 10.1128/JB.182.1.14-22.2000 10613857 PMC94234

[B179] Nakahigashi K Ron EZ Yanagi H Yura T Differential and independent roles of a sigma(32) homolog (RpoH) and an HrcA repressor in the heat shock response of Agrobacterium tumefaciens J Bacteriol 1999 181 7509 7515 10601208 10.1128/jb.181.24.7509-7515.1999 PMC94208

[B180] Thomas JG Baneyx F Roles of the Escherichia coli small heat shock proteins IbpA and IbpB in thermal stress management: comparison with ClpA, ClpB, and HtpG in vivo J Bacteriol 1998 180 5165 5172 9748451 10.1128/jb.180.19.5165-5172.1998 PMC107554

[B181] Clausen T Southan C Ehrmann M The HtrA family of proteases: implications for protein composition and cell fate Mol Cell 2002 10 443 455 10.1016/S1097-2765(02)00658-5 12408815

[B182] Glazebrook J Ichige A Walker GC Genetic analysis of Rhizobium meliloti bacA-phoA fusion results in identification of degP: two loci required for symbiosis are closely linked to degP J Bacteriol 1996 178 745 752 8550509 10.1128/jb.178.3.745-752.1996 PMC177721

[B183] Phillips RW Roop RM Brucella abortus HtrA functions as an authentic stress response protease but is not required for wild-type virulence in BALB/c mice Infect Immun 2001 69 5911 5913 10.1128/IAI.69.9.5911-5913.2001 11500472 PMC98712

[B184] Gomes DF Batista JS Schiavon AL Andrade DS Hungria M Proteomic profiling of Rhizobium tropici PRF 81: identification of conserved and specific responses to heat stress BMC Microbiol 2012 12 84 10.1186/1471-2180-12-84 22647150 PMC3502158

[B185] Nogales J Campos R BenAbdelkhalek H Olivares J Lluch C Sanjuan J Rhizobium tropici genes involved in free-living salt tolerance are required for the establishment of efficient nitrogen-fixing symbiosis with Phaseolus vulgaris Mol Plant Microbe Interact 2002 15 225 232 10.1094/MPMI.2002.15.3.225 11952125

[B186] Bae W Xia B Inouye M Severinov K Escherichia coli CspA-family RNA chaperones are transcription antiterminators Proc Natl Acad Sci USA 2000 97 7784 7789 10.1073/pnas.97.14.7784 10884409 PMC16622

[B187] O'Connell KP Thomashow MF Transcriptional organization and regulation of a polycistronic cold shock operon in Sinorhizobium meliloti RM1021 encoding homologs of the Escherichia coli major cold shock gene cspA and ribosomal protein gene rpsU Appl Environ Microbiol 2000 66 392 400 10.1128/AEM.66.1.392-400.2000 10618253 PMC91835

[B188] Zahran HH Rhizobium-legume symbiosis and nitrogen fixation under severe conditions and in an arid climate Microbiol Mol Biol Rev 1999 63 968 989 10585971 10.1128/mmbr.63.4.968-989.1999 PMC98982

[B189] Domínguez-Ferreras A Muñoz S Olivares J Soto MJ Sanjuán J Role of potassium uptake systems in Sinorhizobium meliloti osmoadaptation and symbiotic performance J Bacteriol 2009 191 2133 2143 10.1128/JB.01567-08 19181803 PMC2655490

[B190] Welsh DT Ecological significance of compatible solute accumulation by micro-organisms: from single cells to global climate FEMS Microbiol Rev 2000 24 263 290 10.1111/j.1574-6976.2000.tb00542.x 10841973

[B191] Fernandez-Aunion C Hamouda TB Iglesias-Guerra F Argandona M Reina-Bueno M Nieto JJ Aouani ME Vargas C Biosynthesis of compatible solutes in rhizobial strains isolated from Phaseolus vulgaris nodules in Tunisian fields BMC Microbiol 2010 10 192 10.1186/1471-2180-10-192 20633304 PMC2918589

[B192] Boncompagni E Østerås M Poggi M-C le Rudulier D Occurrence of choline and glycine betaine uptake and metabolism in the family Rhizobiaceae and their roles in osmoprotection Appl Environ Microbiol 1999 65 2072 2077 10224003 10.1128/aem.65.5.2072-2077.1999 PMC91300

[B193] Dominguez-Ferreras A Soto MJ Perez-Arnedo R Olivares J Sanjuan J Importance of trehalose biosynthesis for Sinorhizobium meliloti osmotolerance and nodulation of alfalfa roots J Bacteriol 2009 191 7490 7499 10.1128/JB.00725-09 19837796 PMC2786593

[B194] McIntyre HJ Davies H Hore TA Miller SH Dufour JP Ronson CW Trehalose biosynthesis in Rhizobium leguminosarum bv. trifolii and its role in desiccation tolerance Appl Environ Microbiol 2007 73 3984 3992 10.1128/AEM.00412-07 17449695 PMC1932737

[B195] Sugawara M Cytryn EJ Sadowsky MJ Functional role of Bradyrhizobium japonicum trehalose biosynthesis and metabolism genes during physiological stress and nodulation Appl Environ Microbiol 2010 76 1071 1081 10.1128/AEM.02483-09 20023090 PMC2820964

[B196] Østerås M Boncompagni E Vincent N Poggi M-C Le Rudulier D Presence of a gene encoding choline sulfatase in Sinorhizobium meliloti bet operon: choline-O-sulfate is metabolized into glycine betaine Proc Natl Acad Sci USA 1998 95 11394 11399 10.1073/pnas.95.19.11394 9736747 PMC21653

[B197] Aktas M Jost KA Fritz C Narberhaus F Choline uptake in Agrobacterium tumefaciens by the high-affinity ChoXWV transporter J Bacteriol 2011 193 5119 5129 10.1128/JB.05421-11 21803998 PMC3187443

[B198] Chen C Beattie GA Characterization of the osmoprotectant transporter OpuC from Pseudomonas syringae and demonstration that cystathionine-beta-synthase domains are required for its osmoregulatory function J Bacteriol 2007 189 6901 6912 10.1128/JB.00763-07 17660277 PMC2045199

[B199] Alloing G Travers I Sagot B Le Rudulier D Dupont L Proline betaine uptake in Sinorhizobium meliloti: characterization of Prb, an Opp-Like ABC transporter regulated by both proline betaine and salinity stress J Bacteriol 2006 188 6308 6317 10.1128/JB.00585-06 16923898 PMC1595395

[B200] Jebbar M Sohn-Bosser L Bremer E Bernard T Blanco C Ectoine-induced proteins in Sinorhizobium meliloti include an ectoine ABC-type transporter involved in osmoprotection and ectoine catabolism J Bacteriol 2005 187 1293 1304 10.1128/JB.187.4.1293-1304.2005 15687193 PMC545623

[B201] Sagot B Gaysinski M Mehiri M Guigonis JM Le Rudulier D Alloing G Osmotically induced synthesis of the dipeptide N-acetylglutaminylglutamine amide is mediated by a new pathway conserved among bacteria Proc Natl Acad Sci USA 2010 107 12652 12657 10.1073/pnas.1003063107 20571117 PMC2906558

[B202] Martinac B Kloda A Evolutionary origins of mechanosensitive ion channels Prog Biophys Mol Biol 2003 82 11 24 10.1016/S0079-6107(03)00002-6 12732265

[B203] Ramachandran VK East AK Karunakaran R Downie JA Poole PS Adaptation of Rhizobium leguminosarum to pea, alfalfa and sugar beet rhizospheres investigated by comparative transcriptomics Genome Biol 2011 12 R106 10.1186/gb-2011-12-10-r106 22018401 PMC3333776

[B204] Bohin J-P Osmoregulated periplasmic glucans in Proteobacteria FEMS Microbiol Lett 2000 186 11 19 10779706 10.1111/j.1574-6968.2000.tb09075.x

[B205] Gil Serrano AM Franco-Rodríguez G González-Jiménez I Tejero-Mateo P Molina JM Dobado JA Megías M Romero MJ The structure and molecular mechanics calculations of the cyclic (1->2)-Beta-d-glucan secreted by Rhizobium tropici CIAT 899 J Mol Struct 1993 301 211 226

[B206] Tanghe A Van Dijck P Thevelein JM Why do microorganisms have aquaporins? Trends Microbiol 2006 14 78 85 10.1016/j.tim.2005.12.001 16406529

[B207] Hernandez-Castro R Rodriguez MC Seoane A Garcia Lobo JM The aquaporin gene aqpX of Brucella abortus is induced in hyperosmotic conditions Microbiology 2003 149 3185 3192 10.1099/mic.0.26678-0 14600230

[B208] Vercruysse M Fauvart M Beullens S Braeken K Cloots L Engelen K Marchal K Michiels J A comparative transcriptome analysis of Rhizobium etli bacteroids: specific gene expression during symbiotic nongrowth Mol Plant Microbe Interact 2011 24 1553 1561 10.1094/MPMI-05-11-0140 21809980

[B209] Vargas Mdel C Encarnacion S Davalos A Reyes-Perez A Mora Y Garcia-delos Santos A Brom S Mora J Only one catalase, katG, is detectable in Rhizobium etli, and is encoded along with the regulator OxyR on a plasmid replicon Microbiology 2003 149 1165 1176 10.1099/mic.0.25909-0 12724378

[B210] Chuchue T Tanboon W Prapagdee B Dubbs JM Vattanaviboon P Mongkolsuk S ohrR and ohr are the primary sensor/regulator and protective genes against organic hydroperoxide stress in Agrobacterium tumefaciens J Bacteriol 2006 188 842 851 10.1128/JB.188.3.842-851.2006 16428387 PMC1347339

[B211] Fontenelle C Blanco C Arrieta M Dufour V Trautwetter A Resistance to organic hydroperoxides requires ohr and ohrR genes in Sinorhizobium meliloti BMC Microbiol 2011 11 100 10.1186/1471-2180-11-100 21569462 PMC3107159

[B212] Barloy-Hubler F Chéron A Hellégouarch A Galibert F Smc01944, a secreted peroxidase induced by oxidative stresses in Sinorhizobium meliloti 1021 Microbiology 2004 150 657 664 10.1099/mic.0.26764-0 14993315

[B213] Bongs G van Pée K-H Enzymatic chlorination using bacterial nonheme haloperoxidases Enzyme Microb Technol 1994 16 53 60 10.1016/0141-0229(94)90109-0

[B214] Wang G Hong Y Johnson MK Maier RJ Lipid peroxidation as a source of oxidative damage in Helicobacter pylori: protective roles of peroxiredoxins Biochim Biophys Acta 2006 1760 1596 1603 10.1016/j.bbagen.2006.05.005 17069977

[B215] Dombrecht B Heusdens C Beullens S Verreth C Mulkers E Proost P Vanderleyden J Michiels J Defence of Rhizobium etli bacteroids against oxidative stress involves a complexly regulated atypical 2-Cys peroxiredoxin Mol Microbiol 2005 55 1207 1221 10.1111/j.1365-2958.2005.04457.x 15686565

[B216] De Smet L Savvides SN Van Horen E Pettigrew G Van Beeumen JJ Structural and mutagenesis studies on the cytochrome c peroxidase from Rhodobacter capsulatus provide new insights into structure-function relationships of bacterial di-heme peroxidases J Biol Chem 2006 281 4371 4379 10.1074/jbc.M509582200 16314410

[B217] Brenot A King KY Janowiak B Griffith O Caparon MG Contribution of glutathione peroxidase to the virulence of Streptococcus pyogenes Infect Immun 2004 72 408 413 10.1128/IAI.72.1.408-413.2004 14688122 PMC344014

[B218] Chiancone E Ceci P The multifaceted capacity of Dps proteins to combat bacterial stress conditions: detoxification of iron and hydrogen peroxide and DNA binding Biochim Biophys Acta 2010 1800 798 805 10.1016/j.bbagen.2010.01.013 20138126

[B219] Ezraty B Aussel L Barras F Methionine sulfoxide reductases in prokaryotes Biochim Biophys Acta 2005 1703 221 229 10.1016/j.bbapap.2004.08.017 15680230

[B220] Crockford AJ Behncke C Williams HD The adaptation of Rhizobium leguminosarum bv. phaseoli to oxidative stress and its overlap with other environmental stress responses Microbiology 1996 142 331 336 10.1099/13500872-142-2-331 33657746

[B221] Shimoda Y Nagata M Suzuki A Abe M Sato S Kato T Tabata S Higashi S Uchiumi T Symbiotic rhizobium and nitric oxide induce gene expression of non-symbiotic hemoglobin in Lotus japonicus Plant Cell Physiol 2005 46 99 107 10.1093/pci/pci001 15668209

[B222] Pullan ST Monk CE Lee L Poole RK Microbial responses to nitric oxide and nitrosative stress: growth, "omic," and physiological methods Methods Enzymol 2008 437 499 519 18433644 10.1016/S0076-6879(07)37025-0

[B223] Poole RK Hughes MN New functions for the ancient globin family: bacterial responses to nitric oxide and nitrosative stress Mol Microbiol 2000 36 775 783 10.1046/j.1365-2958.2000.01889.x 10844666

[B224] Boccara M Mills CE Zeier J Anzi C Lamb C Poole RK Delledonne M Flavohaemoglobin HmpX from Erwinia chrysanthemi confers nitrosative stress tolerance and affects the plant hypersensitive reaction by intercepting nitric oxide produced by the host Plant J 2005 43 226 237 10.1111/j.1365-313X.2005.02443.x 15998309

[B225] Rodionov DA Dubchak IL Arkin AP Alm EJ Gelfand MS Dissimilatory metabolism of nitrogen oxides in bacteria: comparative reconstruction of transcriptional networks PLoS Comput Biol 2005 1 e55 10.1371/journal.pcbi.0010055 16261196 PMC1274295

[B226] Liu L Hausladen A Zeng M Que L Heitman J Stamler JS A metabolic enzyme for S-nitrosothiol conserved from bacteria to humans Nature 2001 410 490 494 10.1038/35068596 11260719

[B227] Montanini B Blaudez D Jeandroz S Sanders D Chalot M Phylogenetic and functional analysis of the Cation Diffusion Facilitator (CDF) family: improved signature and prediction of substrate specificity BMC Genomics 2007 8 107 10.1186/1471-2164-8-107 17448255 PMC1868760

[B228] Rossbach S Mai DJ Carter EL Sauviac L Capela D Bruand C de Bruijn FJ Response of Sinorhizobium meliloti to elevated concentrations of cadmium and zinc Appl Environ Microbiol 2008 74 4218 4221 10.1128/AEM.02244-07 18469129 PMC2446505

[B229] Wiethaus J Wildner GF Masepohl B The multicopper oxidase CutO confers copper tolerance to Rhodobacter capsulatus FEMS Microbiol Lett 2006 256 67 74 10.1111/j.1574-6968.2005.00094.x 16487321

[B230] Cooksey DA Molecular mechanisms of copper resistance and accumulation in bacteria FEMS Microbiol Rev 1994 14 381 386 10.1111/j.1574-6976.1994.tb00112.x 7917425

[B231] Crupper SS Worrell V Stewart GC Iandolo JJ Cloning and expression of cadD, a new cadmium resistance gene of Staphylococcus aureus J Bacteriol 1999 181 4071 4075 10383976 10.1128/jb.181.13.4071-4075.1999 PMC93898

[B232] Nies DH Koch S Wachi S Peitzsch N Saier MH CHR, a novel family of prokaryotic proton motive force-driven transporters probably containing chromate/sulfate antiporters J Bacteriol 1998 180 5799 5802 9791139 10.1128/jb.180.21.5799-5802.1998 PMC107648

[B233] Díaz-Magaña A Aguilar-Barajas E Moreno-Sánchez R Ramírez-Díaz MI Riveros-Rosas H Vargas E Cervantes C Short-chain chromate ion transporter proteins from Bacillus subtilis confer chromate resistance in Escherichia coli J Bacteriol 2009 191 5441 5445 10.1128/JB.00625-09 19581367 PMC2725617

[B234] Páez-Espino D Tamames J de Lorenzo V Cánovas D Microbial responses to environmental arsenic Biometals 2009 22 117 130 10.1007/s10534-008-9195-y 19130261

[B235] Chasteen TG Fuentes DE Tantaleán JC Vásquez CC Tellurite: history, oxidative stress, and molecular mechanisms of resistance FEMS Microbiol Rev 2009 33 820 832 10.1111/j.1574-6976.2009.00177.x 19368559

[B236] Mishra RPN Tisseyre P Melkonian R Chaintreuil C Miché L Klonowska A Gonzalez S Bena G Laguerre G Moulin L Genetic diversity of Mimosa pudica rhizobial symbionts in soils of French Guiana: investigating the origin and diversity of Burkholderia phymatum and other beta-rhizobia FEMS Microbiol Ecol 2012 79 487 503 10.1111/j.1574-6941.2011.01235.x 22093060

[B237] Klonowska A Chaintreuil C Tisseyre P Miché L Melkonian R Ducousso M Laguerre G Brunel B Moulin L Biodiversity of Mimosa pudica rhizobial symbionts (Cupriavidus taiwanensis, Rhizobium mesoamericanum) in New Caledonia and their adaptation to heavy metal-rich soils FEMS Microbiol Ecol 2012 In press 10.1111/j.1574-6941.2012.01393.x 22512707

[B238] Godoy LP Vasconcelos ATR Chueire LMO Souza RC Nicolás MF Barcellos FG Hungria M Genomic panorama of Bradyrhizobium japonicum CPAC 15, a commercial inoculant strain largely established in Brazilian soils and belonging to the same serogroup as USDA 123 Soil Biol Biochem 2008 40 2742 2753

[B239] de la Bastide M McCombie WR Assembling genomic DNA sequences with PHRAP Curr Protoc Bioinformatics 2007 Chapter 11 Unit11 14 18428783 10.1002/0471250953.bi1104s17

[B240] Langmead B Salzberg SL Fast gapped-read alignment with Bowtie 2 Nat Methods 2012 9 357 359 10.1038/nmeth.1923 22388286 PMC3322381

[B241] Gordon D Abajian C Green P Consed: a graphical tool for sequence finishing Genome Res 1998 8 195 202 9521923 10.1101/gr.8.3.195

[B242] Almeida LG Paixao R Souza RC Costa GC Barrientos FJ Santos MT Almeida DF Vasconcelos AT A System for Automated Bacterial (genome) Integrated Annotation–SABIA Bioinformatics 2004 20 2832 2833 10.1093/bioinformatics/bth273 15087310

[B243] Aziz RK Bartels D Best AA DeJongh M Disz T Edwards RA Formsma K Gerdes S Glass EM Kubal M The RAST Server: rapid annotations using subsystems technology BMC Genomics 2008 9 75 10.1186/1471-2164-9-75 18261238 PMC2265698

[B244] Kurtz S Phillippy A Delcher AL Smoot M Shumway M Antonescu C Salzberg SL Versatile and open software for comparing large genomes Genome Biol 2004 5 R12 10.1186/gb-2004-5-2-r12 14759262 PMC395750

[B245] Darling AE Mau B Perna NT ProgressiveMauve: multiple genome alignment with gene gain, loss and rearrangement PLoS One 2010 5 e11147 10.1371/journal.pone.0011147 20593022 PMC2892488

[B246] Eddy SR A new generation of homology search tools based on probabilistic inference Genome Inform 2009 23 205 211 20180275

[B247] Saier MH Jr Tran CV Barabote RD TCDB: the Transporter Classification Database for membrane transport protein analyses and information Nucleic Acids Res 2006 34 D181 186 10.1093/nar/gkj001 16381841 PMC1334385

[B248] Schafer A Tauch A Jager W Kalinowski J Thierbach G Puhler A Small mobilizable multi-purpose cloning vectors derived from the Escherichia coli plasmids pK18 and pK19: selection of defined deletions in the chromosome of Corynebacterium glutamicum Gene 1994 145 69 73 10.1016/0378-1119(94)90324-7 8045426

[B249] Ditta G Stanfield S Corbin D Helinski DR Broad host range DNA cloning system for Gram-negative bacteria: construction of a gene bank of Rhizobium meliloti Proc Natl Acad Sci USA 1980 77 7347 7351 10.1073/pnas.77.12.7347 7012838 PMC350500

[B250] Kovach ME Elzer PH Hill DS Robertson GT Farris MA Roop RM Peterson KM 2nd Four new derivatives of the broad-host-range cloning vector pBBR1MCS, carrying different antibiotic-resistance cassettes Gene 1995 166 175 176 10.1016/0378-1119(95)00584-1 8529885

