# Supplementary figures and images for: Genomic basis of broad host range and environmental adaptability of Rhizobium tropici CIAT 899 and Rhizobium sp. PRF 81 which are used in inoculants for common bean (Phaseolus vulgaris L.)
Source: BMC Genomics. 2012 Dec 27;13:735. doi: 10.1186/1471-2164-13-735 (PMC3557214; doi:10.1186/1471-2164-13-735)

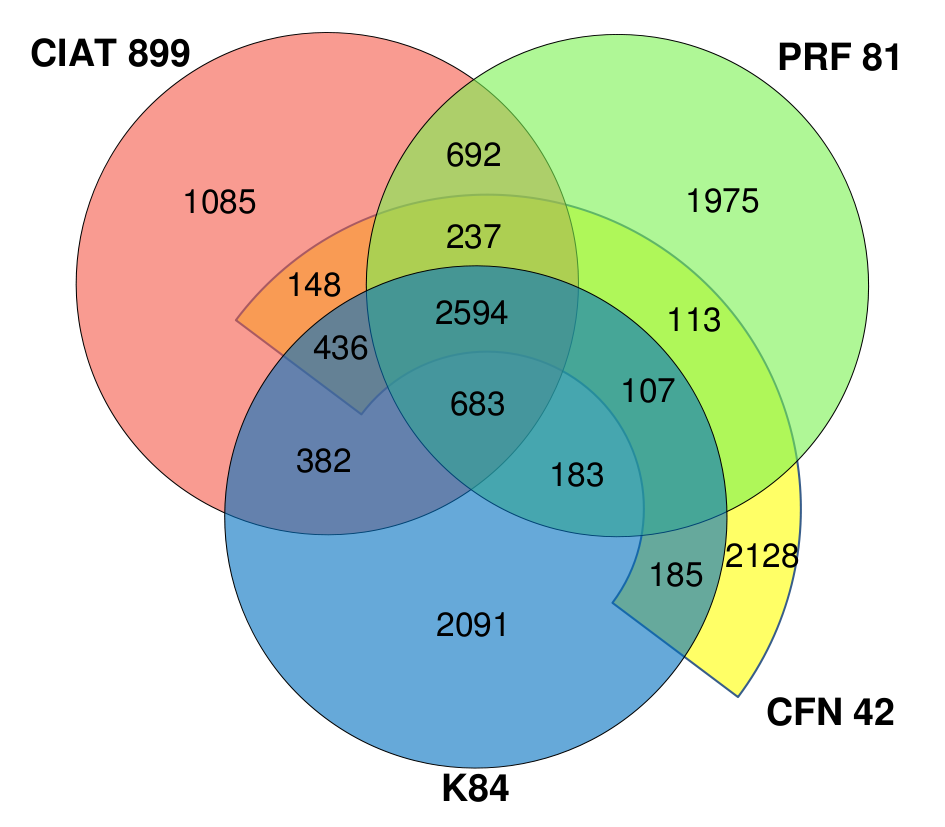

Supplement: Additional file 1 — Diagram showing the number of orthologous gene clusters shared by R. tropici CIAT 899, Rhizobium sp. PRF 81, R. rhizogenes K84 and R. etli CFN 42. Based on SABIA- and RAST-predicted genes. [file 1471-2164-13-735-S1.tiff]

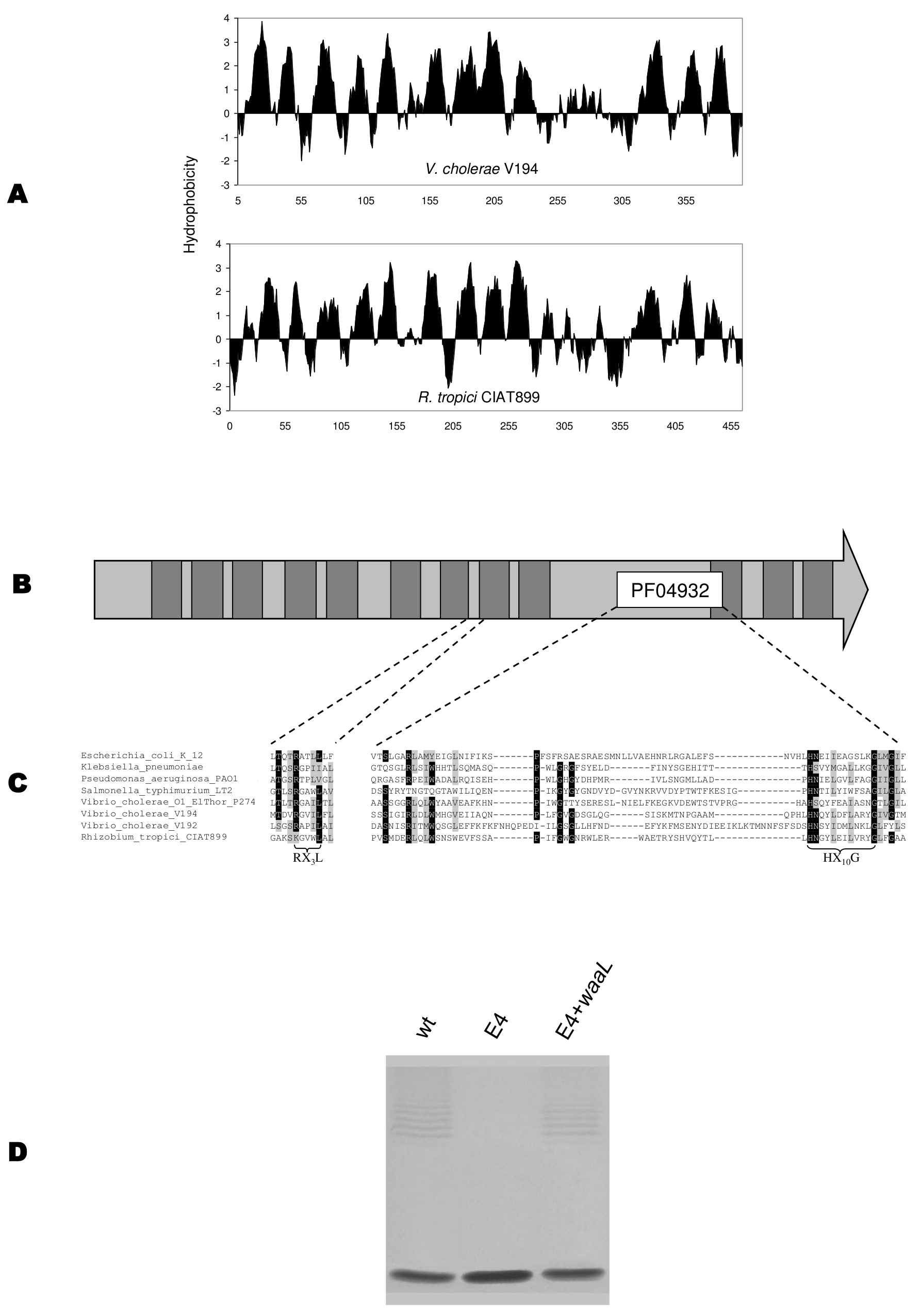

Supplement: Additional file 3 — The WaaL O-antigen ligase of Rhizobium tropici CIAT 899. A, Kite-Dolittle hydrophobicity plots of Vibrio cholerae V194 and R. tropici CIAT 899 WaaL proteins. B, R. tropici CIAT 899 WaaL protein represented as a light gray arrow with predicted membrane-spanning regions indicated with dark grey boxes and the position of the Pfam PF04932 conserved domain indicated with a white box. C, Multiple sequence alignment showing conserved regions shared by WaaL proteins. D, Lipolysaccharide profiles of Rhizobium tropici CIAT 899 (wt), its waaL mutant (E4), and the complemented mutant (E4 + waaL). [file 1471-2164-13-735-S3.tiff]

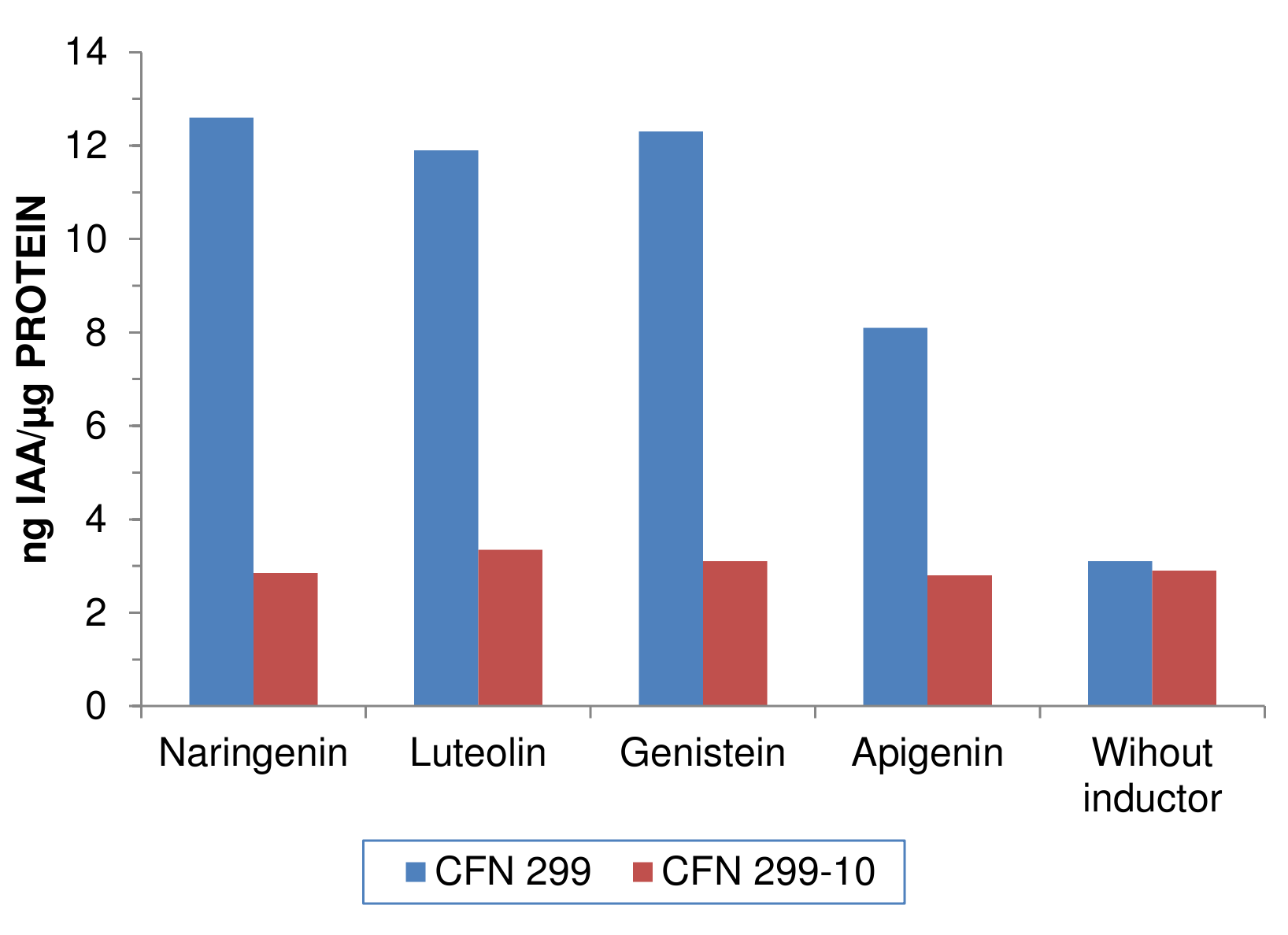

Supplement: Additional file 4 — Induction of indoleacetic acid (IAA) production by flavonoids in R. leucaenae CFN 299 and its 200-kb pSym deletion mutant CFN 299–10. [file 1471-2164-13-735-S4.tiff]
