# Supplementary material for: Genomic basis of broad host range and environmental adaptability of Rhizobium tropici CIAT 899 and Rhizobium sp. PRF 81 which are used in inoculants for common bean (Phaseolus vulgaris L.)
Source: BMC Genomics. 2012 Dec 27;13:735. doi: 10.1186/1471-2164-13-735 (PMC3557214; doi:10.1186/1471-2164-13-735)
Supplement: Additional file 2 — Main characteristics of the three strains in this study. [file 1471-2164-13-735-S2.doc]

**Table S1.** Main characteristics of the three strains in this study.

| Characteristic |  | CIAT 899 | PRF 81 | CFN 299 | Reference |
| --- | --- | --- | --- | --- | --- |
|  |  | Geograpichal origin | | |  |
| Origin |  | Colombia | Brazil | Brazil |  |
|  |  | Genetic | | |  |
|  | Species | *R. tropici* | *Rhizobium* sp | *R. leucaenae* |  |
|  | 72-bp insertion in the *rrs* gene | N | N | Y | 1 |
|  | Fatty acid profile group | IV | IV | III | 1 |
|  | MLSA clustering | IA | isolated | IC | 2 |
|  |  | Morpho-physiological | | |  |
| Growth in/at | pH 4 | + | + | W | 1 |
|  | 40°C | + | + | W | 1 |
|  | LB | + | W | - | 1 |
|  | PY (without Ca) | + | W | - | 1 |
|  | NaCl | + | + | - | 3, this study |
| Melanin synthesis |  | Y | W | N | 3, this study |
| Resistance to | Carbenicillin (50) | + | + | - | 3, this study |
|  | Spectinomycin (100) | + | + | - | 3, this study |
|  | Chloramphenicol (100) | + | + | - | 3, this study |
|  | Rifampin (50) | + | + | - | 3, this study |
|  |  | Symbiotic | | |  |
| Host range^1^ | Isolation host | *P. vulgaris* | *P. vulgaris* | *P. vulgaris* |  |
|  | Broad host range | + | + | + | several |
|  | *Indigofera hirsuta* | + | - | + | 1 |
|  | *Macroptilium atropurpureum* | + | + | + | 1,4 |
|  | *Crotalaria juncea* | + | + | - | 1 |
|  | *Leucaena* spp. | + | + | + | several |
| TLC Nod factor profile |  | 1 | 1 | 1 | 1 |

^1^ None of the strains are able to nodulate *Centrosema pubescens*, *Lupinus albus*, *Medicago sativa*, *Pisum sativum* or *Vicia sativa*.

**References**

1. Hungria M, Andrade DD, Chueire LMD, Probanza A, Guttierrez-Manero FJ, et al. (2000) Isolation and characterization of new efficient and competitive bean (*Phaseolus vulgaris* L.) rhizobia from Brazil. Soil Biol Biochem 32: 1515-1528.

2. Ribeiro RA, Barcellos FG, Thompson FL, Hungria M (2009) Multilocus sequence analysis of Brazilian *Rhizobium* microsymbionts of common bean (*Phaseolus vulgaris* L.) reveals unexpected taxonomic diversity. Res Microbiol 160: 297-306.

3. Martínez-Romero E, Segovia L, Mercante FM, Franco AA, Graham P, et al. (1991) *Rhizobium tropici*, a novel species nodulating *Phaseolus vulgaris* L. beans and *Leucaena* sp. trees. Int J Syst Evol Microbiol 41: 417-426.

4. Hernandez-Lucas I, Segovia L, martinez-Romero E, Pueppke SG (1995) Phylogenetic relationships and host range of *Rhizobium* spp. that nodulate *Phaseolus vulgaris* L. Appl Environ Microbiol 61: 2775-2779.
